# Modeling the Early Phenotype at the Neuromuscular Junction of Spinal Muscular Atrophy Using Patient-Derived iPSCs

**DOI:** 10.1016/j.stemcr.2015.02.010

**Published:** 2015-03-19

**Authors:** Michiko Yoshida, Shiho Kitaoka, Naohiro Egawa, Mayu Yamane, Ryunosuke Ikeda, Kayoko Tsukita, Naoki Amano, Akira Watanabe, Masafumi Morimoto, Jun Takahashi, Hajime Hosoi, Tatsutoshi Nakahata, Haruhisa Inoue, Megumu K. Saito

**Affiliations:** 1 Department of Clinical Application, Center for iPS Cell Research and Application (CiRA), Kyoto University, Kyoto 606-8507, Japan; 2 Department of Cell Growth and Differentiation, Center for iPS Cell Research and Application (CiRA), Kyoto University, Kyoto 606-8507, Japan; 3 Department of Reprogramming Science, Center for iPS Cell Research and Application (CiRA), Kyoto University, Kyoto 606-8507, Japan; 4 Department of Pediatrics, Graduate School of Medical Science, Kyoto Prefectural University of Medicine, Kyoto 602-8566, Japan; 5 Core Research for Evolutional Science and Technology (CREST), Japan Science and Technology Agency, Saitama 332-0012, Japan

## Abstract

Spinal muscular atrophy (SMA) is a neuromuscular disorder caused by mutations of the *survival of motor neuron 1* (*SMN1*) gene. In the pathogenesis of SMA, pathological changes of the neuromuscular junction (NMJ) precede the motor neuronal loss. Therefore, it is critical to evaluate the NMJ formed by SMA patients’ motor neurons (MNs), and to identify drugs that can restore the normal condition. We generated NMJ-like structures using MNs derived from SMA patient-specific induced pluripotent stem cells (iPSCs), and found that the clustering of the acetylcholine receptor (AChR) is significantly impaired. Valproic acid and antisense oligonucleotide treatment ameliorated the AChR clustering defects, leading to an increase in the level of full-length SMN transcripts. Thus, the current in vitro model of AChR clustering using SMA patient-derived iPSCs is useful to dissect the pathophysiological mechanisms underlying the development of SMA, and to evaluate the efficacy of new therapeutic approaches.

## Highlights

• A pluripotent cell model of NMJ pathology in spinal muscular atrophy was established
• SMA iPSC-derived motor neurons from SMA patients failed to cluster AChRs
• SMA iPSC-derived motor neurons accumulated neurofilament proteins in distal axons
• VPA and antisense oligonucleotide ameliorated the NMJ pathology

## Graphical Abstract

**Figure:**
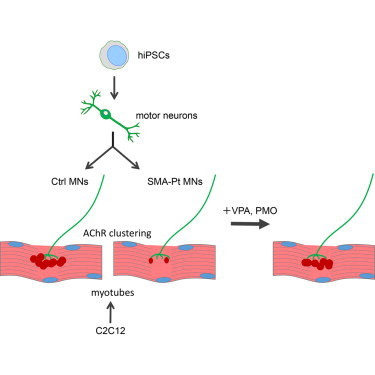

## Teaser

In this article, Saito and colleagues established an in vitro model of NMJ formation using human iPSC-derived motor neurons. They found that SMA patient-derived iPSCs failed to cluster acetylcholine receptors in the model. They also showed that this model was useful to evaluate the effect of potential therapeutic approaches on NMJ pathology.

## Introduction

Proximal spinal muscular atrophy (SMA) is an autosomal recessive neuromuscular disorder caused by the homozygous deletion or mutation of the *survival of motor neuron 1* (*SMN1*) gene, resulting in a deficiency of the ubiquitously expressed SMN protein. Patients suffer from progressive muscular weakness, which eventually results in respiratory failure in severe case. Since there are no effective treatment options, SMA remains the most frequent genetic cause of infant mortality (Burghes and Beattie, 2009; Lefebvre et al., 1995). *SMN2*, a unique gene in humans, is an almost identical copy gene of *SMN1*, but has a constitutive C to T transition in its exon 7. This transition affects the splicing of *SMN2* mRNA, thereby resulting in the predominant production of a shorter unstable isoform termed SMN-Δ7 (Monani et al., 1999). Although *SMN2* is unable to compensate for the homozygous loss of *SMN1* because of the lower amount of full-length SMN transcripts (SMN-FL), the copy number of *SMN2* affects the severity of SMA (McAndrew et al., 1997).

Based on clinical examinations and the pathological analyses of end-stage specimens, SMA historically has been described as a lower motor neuron (MN) disease characterized by the degeneration of the anterior horn cells of the spinal cord, which subsequently leads to skeletal muscle atrophy and weakness (Dubowitz, 2009). However, recent studies in SMA animal models have shown that the earliest detectable pathological change is observed at the neuromuscular junctions (NMJs), including neurofilament (NF) accumulation at the endplate on postnatal day 1 (Ling et al., 2012). Subsequently, a central synaptic defect is observed on day 4, motor neuronal loss manifests around day 9, and almost all mice die by day 15 (Sleigh et al., 2011). Therefore, impairment of the NMJ structure appears to be one of the most important phenotypes, and the development of agents that target the NMJ pathology may represent an attractive approach for therapy. Indeed, an aberrant ultrastructure of NMJs also has been reported in a human prenatal specimen obtained from a fetus with type I SMA (Martínez-Hernández et al., 2013).

Despite recent advances in our understanding of the disease, the detailed mechanism(s) involved in the NMJ formation and maturation, which occur during both the prenatal and early postnatal periods, have not been fully described (Wu et al., 2012). With a few exceptions, the analyses of the pathological roles of SMN have been conducted mainly using animal models, because there are difficulties associated with obtaining human specimens from either biopsy or post-mortem samples. Although there are several available transgenic mouse models of SMA, inter-species differences between mice and humans, such as the existence of *SMN2* in humans, hamper the translation of the findings in mouse studies to human clinical trials (Harahap et al., 2012; Martínez-Hernández et al., 2009; Park et al., 2010). Furthermore, there are difficulties related to evaluating the pathological roles of neurons and myocytes separately. To establish a platform to elucidate the pathology of the NMJ in SMA patients, we herein evaluated the ability of MNs from SMA patient-derived induced pluripotent stem cells (iPSCs) (Takahashi et al., 2007) to form NMJs.

## Results

### Generation and Characterization of iPSCs from Type 1 SMA Patients

**Figure 1:**
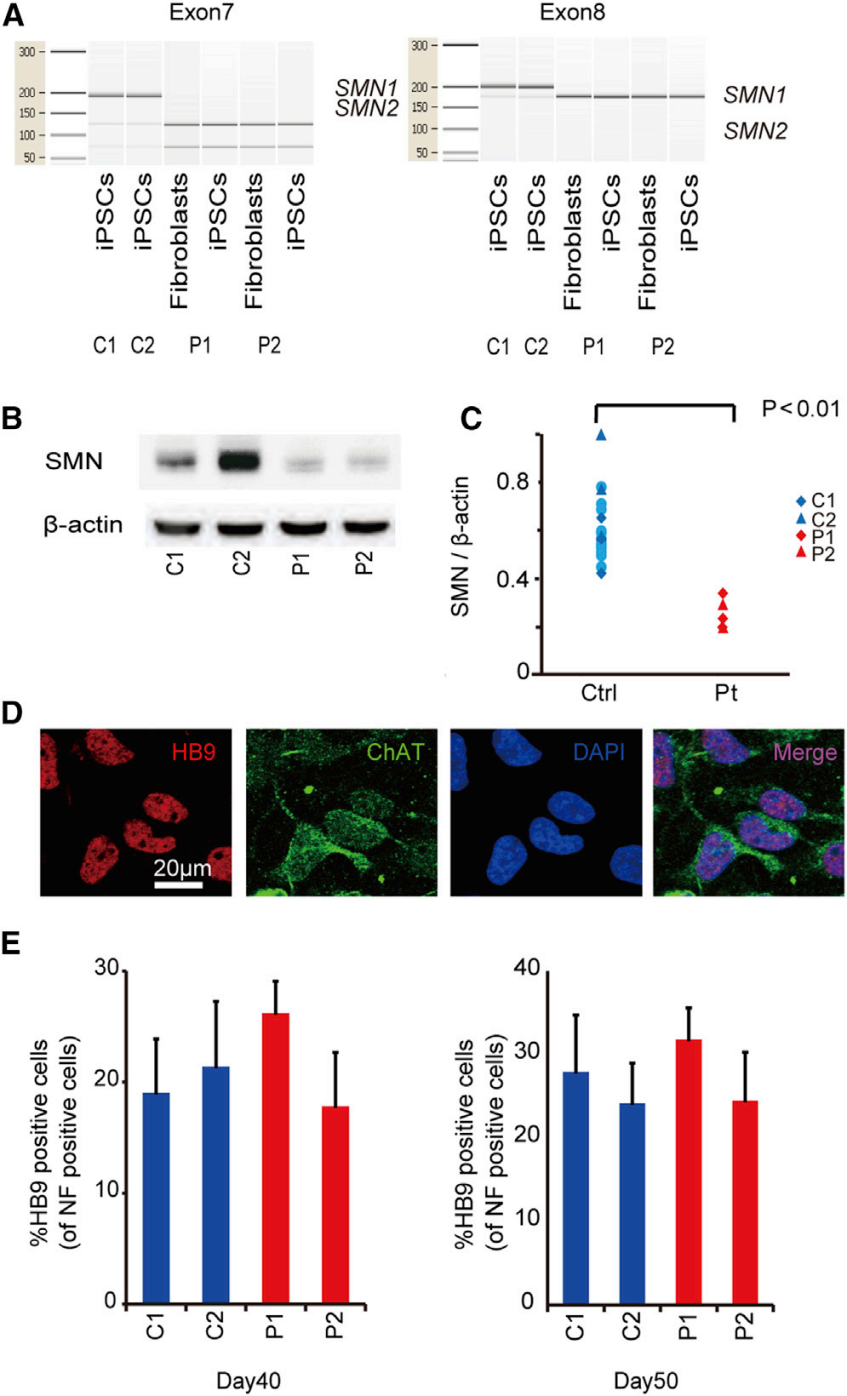
Differentiation of iPSCs into Spinal MNs (A) The PCR restriction fragment-length polymorphism (RFLP) analysis using a bioanalyzer confirmed that the SMA-iPSCs maintained exon 7 and 8 deletions in the *SMN1* gene. (B) Western blot analysis of SMN proteins. (C) Quantification of the SMN protein expression relative to that of β-actin (eight control PSC clones and two SMA-iPSC clones) (n = 3, Wilcoxon rank-sum test). (D) Immunostaining of SMA-iPSC-derived MNs. HB9, red; and ChAT, green on day 60. (E) The quantitative immunocytochemical analysis for HB9-positive iPSC-derived MNs (means ± SEM, n = 3). See also Figure S1.

Fibroblasts from two independent type 1 SMA patients (Coriell IDs GM00232 and GM03813, referred to as P1 and P2) were reprogrammed by episomal vectors (Okita et al., 2011). Both SMA-iPSC clones used in this study (P1 and P2) showed a characteristic human embryonic stem cell (ESC)-like morphology, and expressed pluripotent markers compared to control ESC (KhES1) and iPSCs (201B7 and 409B2, referred to as C1 and C2) (Figures S1A and S1B). The RNA microarray analysis confirmed that the global gene expression pattern (Figures S1C and S1D) and levels of pluripotent stem cell-related genes (Figure S1E) in the P1 and P2 iPSCs were similar to that observed in the control iPSCs. The P1 and P2 iPSCs also exhibited demethylation of NANOG and OCT3/4 loci (Figure S1F) and maintained a normal karyotype (Figure S1G). Pluripotency of P1 and P2 iPSC lines were confirmed by teratoma formation assay (Figure S1H). The expression of introduced transgenes was rarely detected (Figure S1I). The genetic identity of the iPSC clones was proven by a short tandem repeat analysis (data not shown). SMA-iPSCs were confirmed to carry homozygosis deletions of exons 7 and 8 of the *SMN1* gene (Figure 1A; van der Steege et al., 1995), and their SMN protein level was also significantly lower than that in control iPSCs, including C1 and C2 (Figures 1B and 1C).

### MN Differentiation of SMA-iPSCs

We next directed the SMA- and control iPSCs to differentiate into MNs using a previously reported cortical neuron (Morizane et al., 2011) and spinal MN differentiation protocol, with some modifications (Egawa et al., 2012). The iPSC-derived neurons expressed neuronal markers (Figure S2A) and MN-specific markers (Figure 1D). The expression of the introduced transgene *OCT3/4* detected in the P1-iPSCs was completely silenced after 40 days of MN differentiation (Figure S2B). Although a significant decline in MNs over time has been reported as a hallmark of SMA patient iPSC-derived MNs (Chang et al., 2011; Corti et al., 2012; Ebert et al., 2009), the two independent SMA-iPSC lines produced and maintained a similar number of HB9-positive MNs compared to control iPSCs after 40 and 50 days of differentiation (Figure 1E). Therefore, in our MN differentiation system, the SMA-iPSC lines were competent in generating mature MNs and presented no evidence of cell-autonomous MN loss by 50 days of differentiation.

### Formation of NMJ-like Structure with SMA-iPSCs

**Figure 2:**
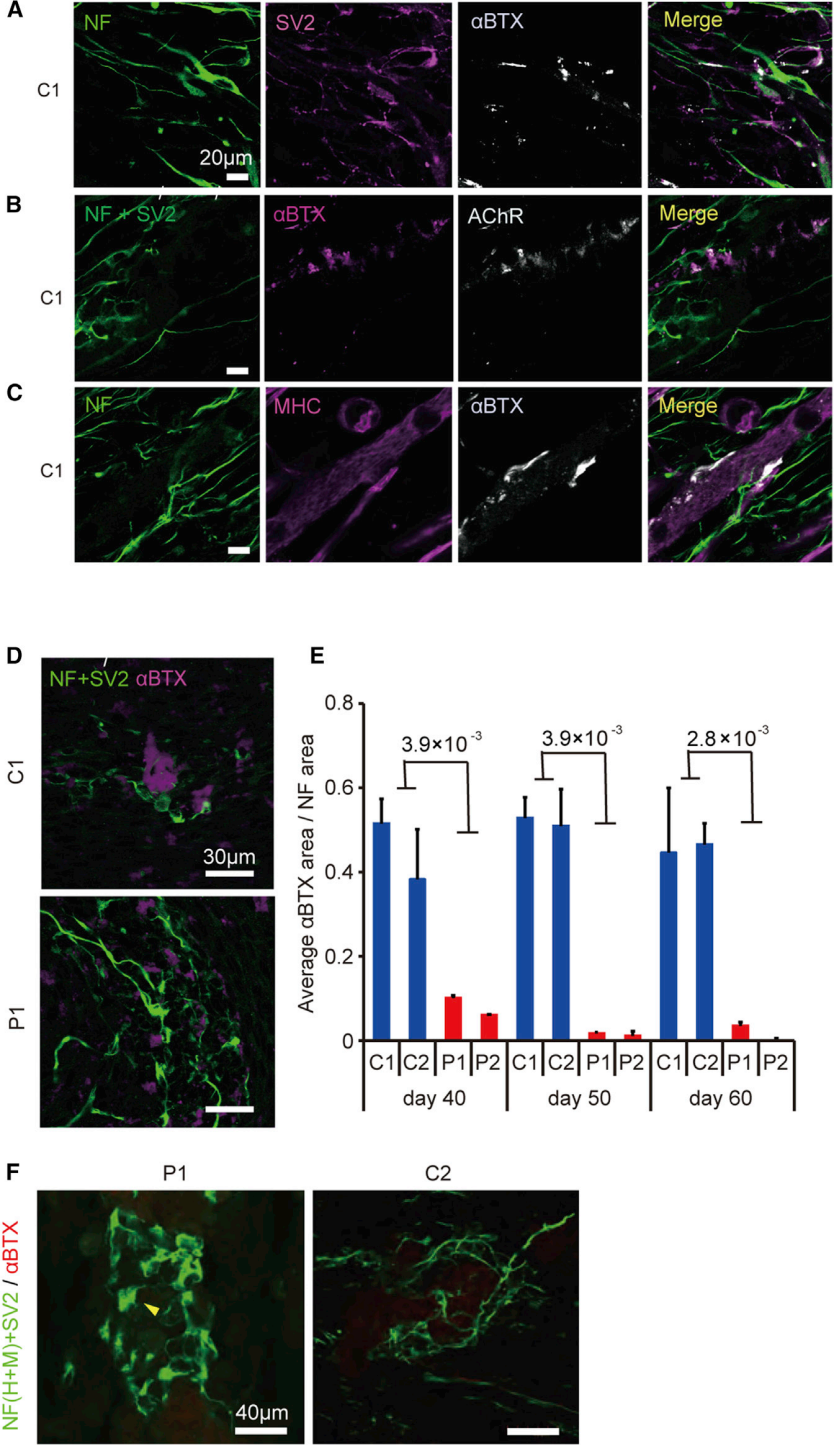
The Pre- and Post-synaptic Morphological Defects in Type 1 SMA (A–D) Representative confocal micrographs showing the immunocytochemically labeled NMJ-LSs of iPSC-derived neurons and C2C12 myotubes. (A and B) Representative images of AChR clusters formed by C1 MNs. (C) AChR clusters stained with MHC. (D) NMJ-LSs of patient (P1)- and control (C1)-derived MNs on day 60. (E) Quantitative immunocytochemical analysis of the α-BTX-positive area (means ± SEM, n = 3, Wilcoxon rank-sum test). (F) Abnormal NF accumulation (yellow arrows) and poor terminal arborization of MNs in the SMA NMJ-LSs. See also Figure S2.

We next tried to develop an in vitro NMJ formation model using the human iPSC-derived MNs. We co-cultured control MNs with differentiated murine C2C12 cell lines and found that αBTX-positive AChRs were clustered at the site of SV2-positive neuronal endplates (Figure 2A). To exclude the presence of unexpected artifacts of αBTX staining under these conditions, we co-stained samples with αBTX and anti-AChR antibodies and confirmed that the regions of both positive staining merged completely (Figure 2B). In addition, the AChR clusters were localized on myosin heavy chain (MHC)-positive multinuclear myotubes (Figure 2C).

We next evaluated the AChR clustering on myotubes co-cultured with SMA-iPSC-derived MNs and found it remarkably impaired (Figure 2D). We evaluated the area of αBTX to assess the ability of MNs to form and maintain the NMJ-like structures (NMJ-LSs). We evaluated the AChR clustering at several time points to determine whether MN maturation affects the phenotype of the NMJ-LSs. The SMA-iPSC-derived MNs induced far less AChR clustering in myotubes than control iPSC-derived MNs did at 40, 50, or 60 days of differentiation (Figures 2E and S2C). AChR clustering was rarely observed for either the SMA- or control iPSC-derived MNs at time points earlier than day 30 (data not shown). We also evaluated the average size of each AChR cluster (Figure S2D) and number of AChR clusters (Figure S2E). Consequently, the AChR clusters formed with SMA-iPSC-derived MNs were smaller and fewer in number than those formed with controls. To evaluate whether co-culturing with MNs affects the maturation status of C2C12, we compared the expression levels of embryonic (*Myh3*) and perinatal (*Myh8*) subtypes of MHCs in skeletal muscle with or without co-culturing (Stern-Straeter et al., 2011; Figure S2F). However, the ratio of expression of these genes was not different, indicating a lack of difference in the maturation of C2C12.

Although motor neuronal loss was not observed in our MN differentiation system without co-culture, there remains a possibility that the NMJ defects in SMA patient-derived MNs are due to MN death occurring under the co-culture conditions. To exclude the possibility, we performed TUNEL staining of the MNs (Figures S2G and S2H). The number of TUNEL-positive apoptotic MNs did not increase during co-culturing, which confirms that synapse loss indeed occurs in surviving MNs. The accumulation of NF proteins and poor arborization in distal axons and motor nerve terminals are considered to be specific features of SMA model mice, although their significance in the pathogenesis of human SMA is unknown (Cifuentes-Diaz et al., 2002; Kariya et al., 2008; Kong et al., 2009). These findings were observed in SMA-iPSC-derived MNs (Figure 2F), which indicates the functional deficit of the motor endplate in SMA. Taken together, the SMA-iPSC-derived MNs had impaired AChR clustering on myotubes in the absence of MN loss, indicating that there was functional impairment of MNs in terms of their forming or allowing for the maturation of NMJs.

### The SMA Phenotype in NMJ-LS Was Rescued by Valproic Acid and Phosphorodiamidate Morpholino Oligonucleotides

**Figure 3:**
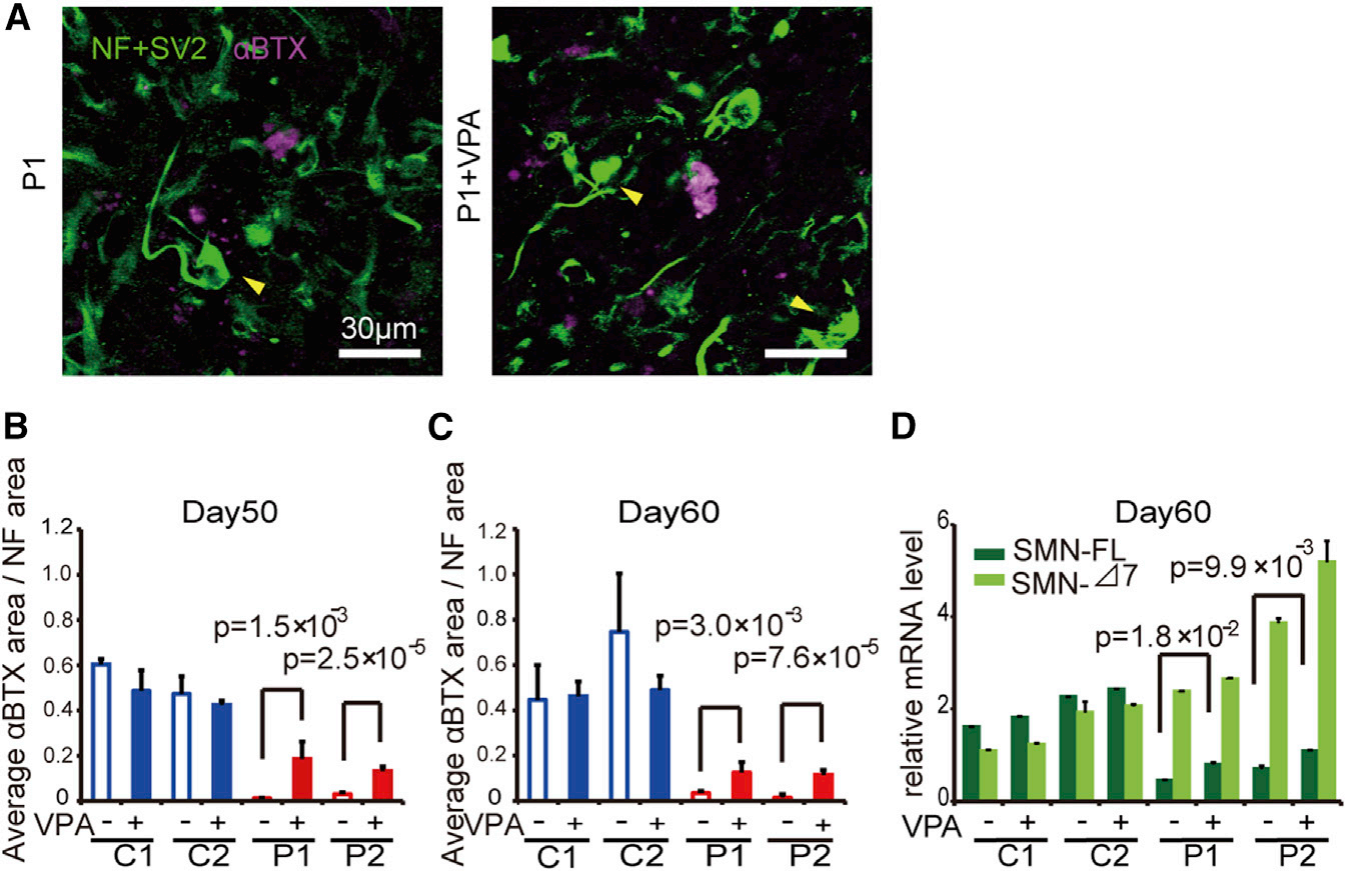
VPA Treatment Rescues the NMJ Pathology in SMA Patient-Derived Cell Cultures (A) Representative images of NMJ-LSs formed with or without VPA. Yellow arrows indicate abnormal NF accumulation. (B and C) Quantitative immunocytochemical analysis of the α-BTX area after VPA treatment (1 mM) (means ± SEM, n = 3, Student’s t test). (D) The mRNA levels of SMN-FL and SMN-Δ7 in SMA-iPSC MNs (means ± SEM, n = 3, Student’s t test). See also Figure S3 and Tables S1 and S2.

Since the loss of NMJ formation is regarded to be an important hallmark preceding the motor neuronal loss, compounds that ameliorate the NMJ pathology may serve as promising therapeutic drug candidates. To evaluate whether the NMJ-LSs formation system used in our experiments can serve as a prototype for evaluating drug candidates, we assessed whether the SMN-inducing drug, valproic acid (VPA), could increase the AChR clustering in our co-culture system. VPA is known to increase the functional SMN protein by activating various promoters, including that of *SMN2*, and by correcting the abnormal splicing of *SMN2* exon 7, mainly through the upregulation of splicing factors (Harahap et al., 2012). Co-culturing myotubes and SMA-iPSC-derived MNs treated with VPA significantly increased the AChR clustering (Figures 3A–3C and S3A), while the clustering was not induced when monocultured myotubes were treated with VPA (data not shown). NF accumulation was not rescued by VPA treatment (Figure 3A). We confirmed that VPA treatment increased both the SMN-Δ7 and SMN-FL mRNA levels in the SMA-iPSC-derived MNs (Figure 3D).

We next performed an RNA sequencing (RNA-seq) analysis to evaluate the effects of VPA on the expression profiles of the MNs, and we found 227 genes that were upregulated more than 2-fold in the VPA-treated MNs, whereas 51 genes were downregulated (Figure S3B; Tables S1 and S2). The expression levels of splicing factors known to be affected by VPA treatment (Brichta et al., 2003; Harahap et al., 2012) were slightly upregulated as reported, in both the control and patient-derived MNs (Figure S3C).

**Figure 4:**
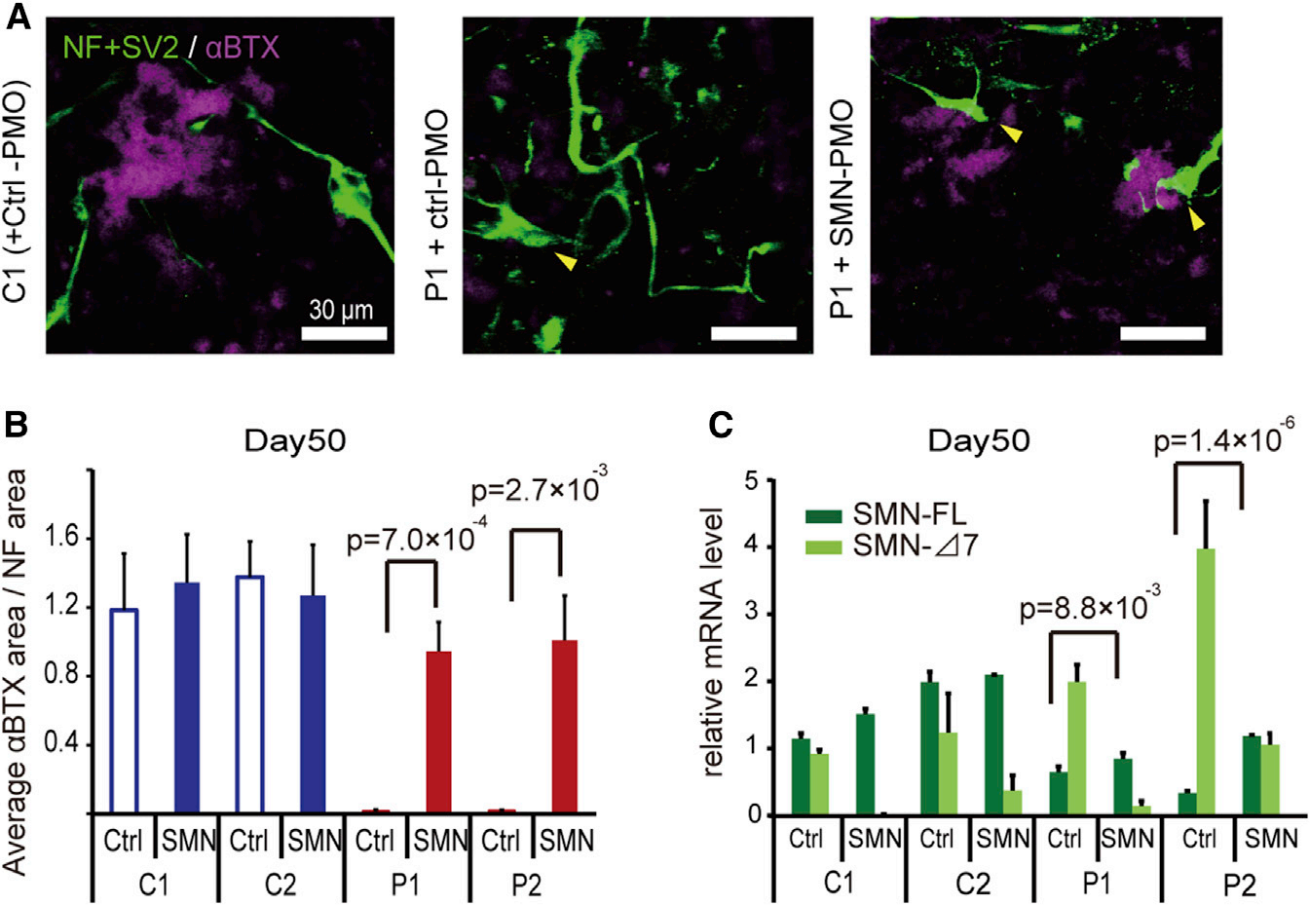
PMO Treatment Rescues the NMJ Pathology in SMA Patient-Derived Cell Cultures (A) Representative images of NMJ-LSs formed with or without PMOs. Yellow arrows indicate abnormal NF accumulation. (B) Quantitative immunocytochemical analysis of the α-BTX area after PMO treatment (means ± SEM, n = 3, Student’s t test). (C) The mRNA levels of SMN-FL and SMN-Δ7 with PMO treatment (means ± SEM, n = 3, Student’s t test). See also Figure S3.

To further explore the validity of NMJ-LSs, we next evaluated the effects of splicing modification on *SMN2*. Recently, the application of antisense oligonucleotides to promote *SMN2* exon 7 retention has been proposed as an alternative therapeutic approach for SMA (Mitrpant et al., 2013). For this purpose, we introduced phosphorodiamidate morpholino oligonucleotides (PMOs) targeting the intronic silencing motif in *SMN2* intron 7. Consequently, the SMN-specific PMO treatment dramatically improved AChR clustering with the patient-derived MNs (Figures 4A, 4B, and S3D). The PMO treatment also recovered the expression of SMN-FL (Figure 4C) and improved, at least partially, the abnormal NF accumulation (Figure 4A). We consider that these data indicate the potential therapeutic advantages of PMO for SMA patients. Overall, the NMJ-LS morphology could be useful for evaluating new therapeutic approaches for SMA.

## Discussion

Previous studies regarding the phenotype of MNs differentiated from SMA-iPSCs have focused mainly on the cell autonomous defects, such as shortened neurite extension, and the reduced size of the cell body; but, their significance in relation to the in vivo phenotype remains unclear (Chang et al., 2011; Ebert et al., 2009). Although motor neuronal loss during culture also has been reported, this is not observed in vivo until the end stage of the disease. We did not observe any progressive motor neuronal loss during culture, even during the longer time period, which is contrary to the previous reports (Chang et al., 2011; Corti et al., 2012; Ebert et al., 2009; Sareen et al., 2012). The precise reason for this difference is unknown, but a variety of methodological differences during culture, such as difference in the timing of the analysis, the protocol of MN differentiation, and the methods used for the evaluation, could all have contributed to this phenotypic variation. Although Corti et al. briefly reported the detection of NMJ defects after co-culturing human myoblasts and SMA-iPSC-derived MNs in a recent report (Corti et al., 2012), MN loss was observed without co-culturing the cells with myotubes in their SMA model, leaving the possibility that preceding motor neuronal death may have led to the defect in forming the NMJ. In contrast, the patient-derived iPSCs used in our study yielded a similar ratio of HB9+ MNs compared to the two control lines. Moreover, we observed a significant reduction in AChR clustering, with no significant increases in the number of TUNEL-positive MNs, during co-culturing.

Since we focused on the pathology of NMJ, we performed a detailed examination of our NMJ-LS. To evaluate whether the developmental status of the MNs affects the NMJ pathology, we evaluated AChR clustering at three different time points and obtained consistent data. In addition to impaired AChR clustering, we detected abnormal presynaptic NF accumulation at the endplate in the SMA-iPSC-derived neurons, which also indicates that synaptic breakdown precedes motor neuronal death in our model. These data support a hypothesis that MNs derived from patient iPSCs are a major contributing factor to the pathogenesis in the NMJ due to SMA. Based on these observations, we considered that the morphological defect of NMJ-LS in our culture was due to functional impairments of the MNs in target pathfinding and/or in inducing or maintaining AChR clustering, rather than due to motor neuronal loss. Considering that the formation and maintenance of NMJs has been indicated to precede the occurrence of MN death even in humans, as mentioned above, the vulnerability of MNs in SMA patients seems to be due not only to the autonomous cell susceptibility to various stresses, but also as a consequence of the NMJ defect, which causes the impairment of neurotrophic factors and subsequent death of MNs (Fidziańska and Rafalowska, 2002; Fischer et al., 2004).

In summary, we demonstrated that the early SMA phenotype in NMJ could be recapitulated with MN differentiated from SMA-iPSCs. Since the available outcome measures to assess the drug efficacy in SMA are limited, our findings that the NMJ is a vulnerable target amenable to rescue by VPA and PMOs seems to indicate that this system will be useful for future evaluations of novel therapeutic candidates. Further experiments with patient-derived iPSCs on the neurodevelopmental aspects of the neuromuscular system, including specific molecular and cellular functions of SMN in both muscle and MN, will provide new insights into the pathophysiology of SMA. We believe that this approach will also help deepen our understanding of the pathogenesis of the muscle and MN interactions on the formation of the NMJ.

## Experimental Procedures

### Study Approval

Use of human ESCs was approved by the Ministry of Education, Culture, Sports, Science and Technology of Japan (MEXT). The study plan for recombinant DNA research has been approved by the recombinant DNA experiments safety committee of Kyoto University. An experimental protocol was approved by the Animal Research Committee of CiRA, Kyoto University.

### MN Differentiation and Co-culture with C2C12

The iPSCs were dissociated into single cells and quickly re-aggregated in DFK 5% medium (DMEM/F12 medium supplemented with KSR, NEAA, 2-mercaptoethanol, L-Glutamate, SB431542, dorsomorphin, and Y27632) (9,000 cells/150 μl/well) using 96-well low cell-adhesion plates (Lipidure-coat U96w from Nunc) (Eiraku et al., 2008; Morizane et al., 2011). From day 8, the cell aggregates were treated with Sonic hedgehog (100 ng/ml) and retinoic acid (1 μM) for 1 week (Wada et al., 2009). On day 20, the cell aggregates were plated onto poly-l-lysin/laminin-coated culture dishes in neuronal medium (neurobasal medium [Gibco] supplemented with the neurotrophic factors GDNF, BDNF, and NT3 [10 ng/ml, R&D Systems]). The medium was changed every 3 to 4 days thereafter.

For the co-culture with neuronal cells, the fusion of C2C12 myoblasts was induced by switching to the differentiation medium (DMEM supplemented with horse serum). On day 4, the MNs that had differentiated from the iPSCs (differentiation days 34–54) were harvested and plated on the induced myotubes, and the medium was changed to neuronal medium. Thereafter, the cultures were fed every 2 days by changing half of the medium.

### VPA Treatment

Co-cultured samples were treated with or without 1 mM VPA by changing half of the medium every 2 days. After 6 days of drug treatment, the area of NF and αBTX immunostaining was detected by immunocytochemistry and was analyzed by the IN Cell Analyzer 2000 software program.

### PMO Treatment

Designed PMOs SMN2E7D(-10-29) for suppressing splice silencing motifs in intron 7 of *SMN2* (Mitrpant et al., 2013) and its negative control were purchased from Gene Tools. SMN- or Ctrl-PMO (10 μM in medium) were introduced with the Endo-Porter (Gene Tools) on day 1 of co-culturing, and the cells were subsequently cultured for 3 days.

### Statistics

Statistic functions in Microsoft Excel 2013 were used for statistical analyses. Statistical significance was determined using Student’s t test and Wilcoxon rank-sum test, p < 0.05 was considered significant, and n represents the number of independent experiments.

## Author Contributions

M.Yo., S.K., M.M., H.H., J.T., T.N., H.I., and M.K.S. designed the research. M.Yo., S.K., N.E., M.Ya., R.I., N.A., and K.T. performed the research. M.Yo., A.W., M.Ya., and R.I. analyzed the data. M.Yo. and M.K.S. wrote the paper.

## Supplemental Information

Document S1. Supplemental Experimental Procedures, Figures S1–S3, and Tables S1–S3

Document S2. Article plus Supplemental Information
