# Supplementary material for: Modeling the Early Phenotype at the Neuromuscular Junction of Spinal Muscular Atrophy Using Patient-Derived iPSCs
Source: Stem Cell Reports. 2015 Mar 19;4(4):561–8. doi: 10.1016/j.stemcr.2015.02.010 (PMC4400613; doi:10.1016/j.stemcr.2015.02.010)
Supplement: Document S1. Supplemental Experimental Procedures, Figures S1–S3, and Tables S1–S3 [file mmc1.pdf]

Stem Cell Reports, Volume *4*

Supplemental Information

Modeling the Early Phenotype at the Neuromuscular Junction of Spinal Muscular Atrophy Using Patient-Derived iPSCs

Michiko Yoshida, Shiho Kitaoka, Naohiro Egawa, Mayu Yamane, Ryunosuke Ikeda, Kayoko Tsukita, Naoki Amano, Akira Watanabe, Masafumi Morimoto, Jun Takahashi, Hajime Hosoi, Tatsutoshi Nakahata, Haruhisa Inoue, and Megumu K. Saito

Figure S1

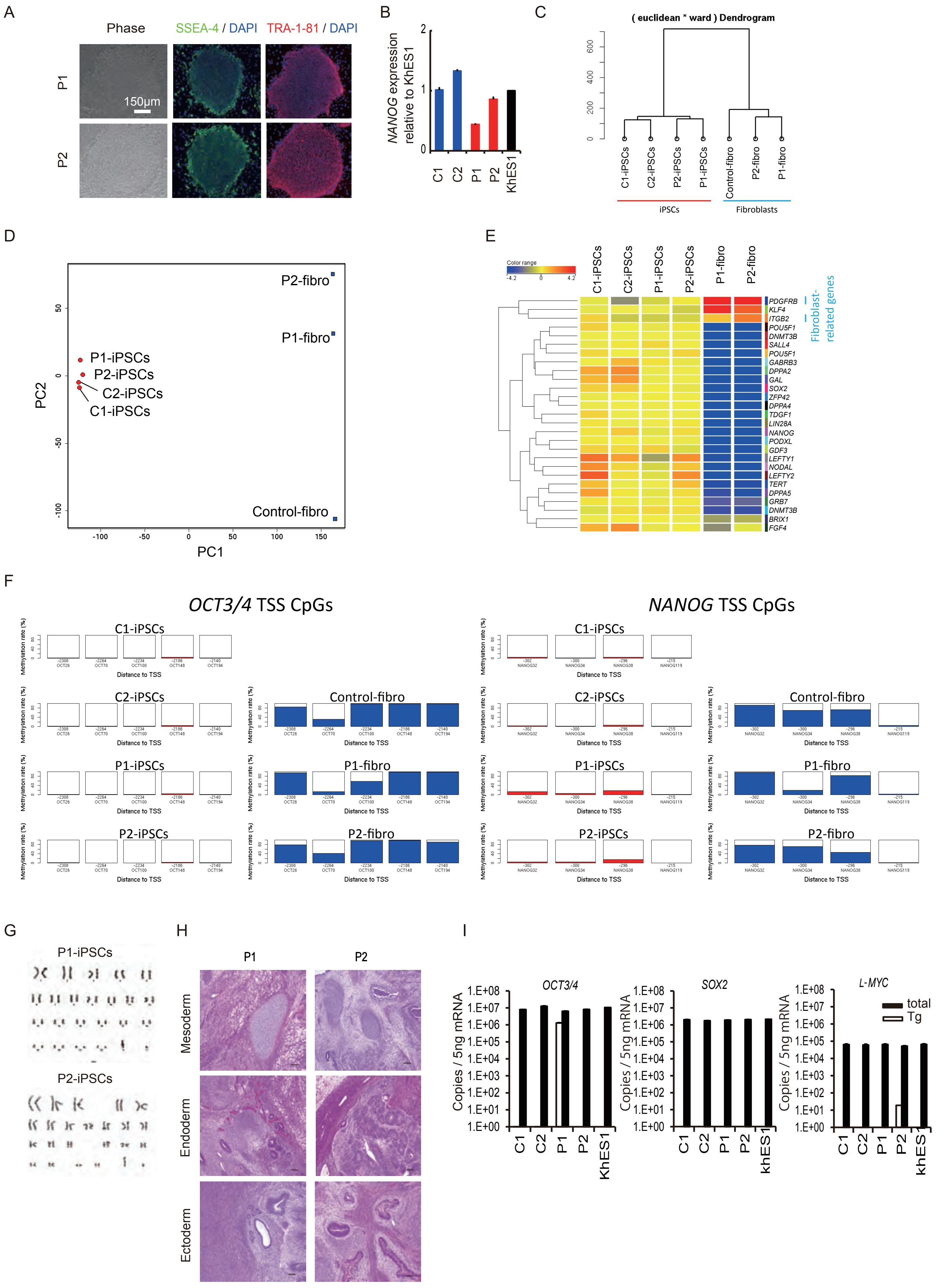
A
Phase
SSEA-4 / DAPI
TRA-1-81 / DAPI
P1
P2
150µm
B
NANOG expression relative to KhES1
C1
C2
P1
P2
KhES1
C
(euclidean * ward) Dendrogram
C1-iPSCs
C2-iPSCs
P2-iPSCs
P1-iPSCs
Control-fibro
P2-fibro
P1-fibro
iPSCs
Fibroblasts
D
PC1
PC2
P2-fibro
P1-fibro
P1-iPSCs
P2-iPSCs
C2-iPSCs
C1-iPSCs
Control-fibro
E
Color range
C1-iPSCs
C2-iPSCs
P1-iPSCs
P2-iPSCs
P1-fibro
P2-fibro
PDGFRB
KLF4
ITGB2
POU5F1
DNMT3B
SALL4
POU5F1
GABRB3
DPPA2
GAL
SOX2
ZFP42
DPPA4
TDGF1
LIN28A
NANOG
PODXL
GDF3
LEFTY1
NODAL
LEFTY2
TERT
DPPA5
GRB7
DNMT3B
BRIX1
FGF4
Fibroblast-related genes
F
OCT3/4 TSS CpGs
NANOG TSS CpGs
C1-iPSCs
C2-iPSCs
P1-iPSCs
P2-iPSCs
Control-fibro
P1-fibro
P2-fibro
Methylation rate (%)
Distance to TSS
G
P1-iPSCs
P2-iPSCs
H
P1
P2
Mesoderm
Endoderm
Ectoderm
I
OCT3/4
SOX2
L-MYC
Copies / 5ng mRNA
total
Tg
C1
C2
P1
P2
KhES1
khES1

Figure S2

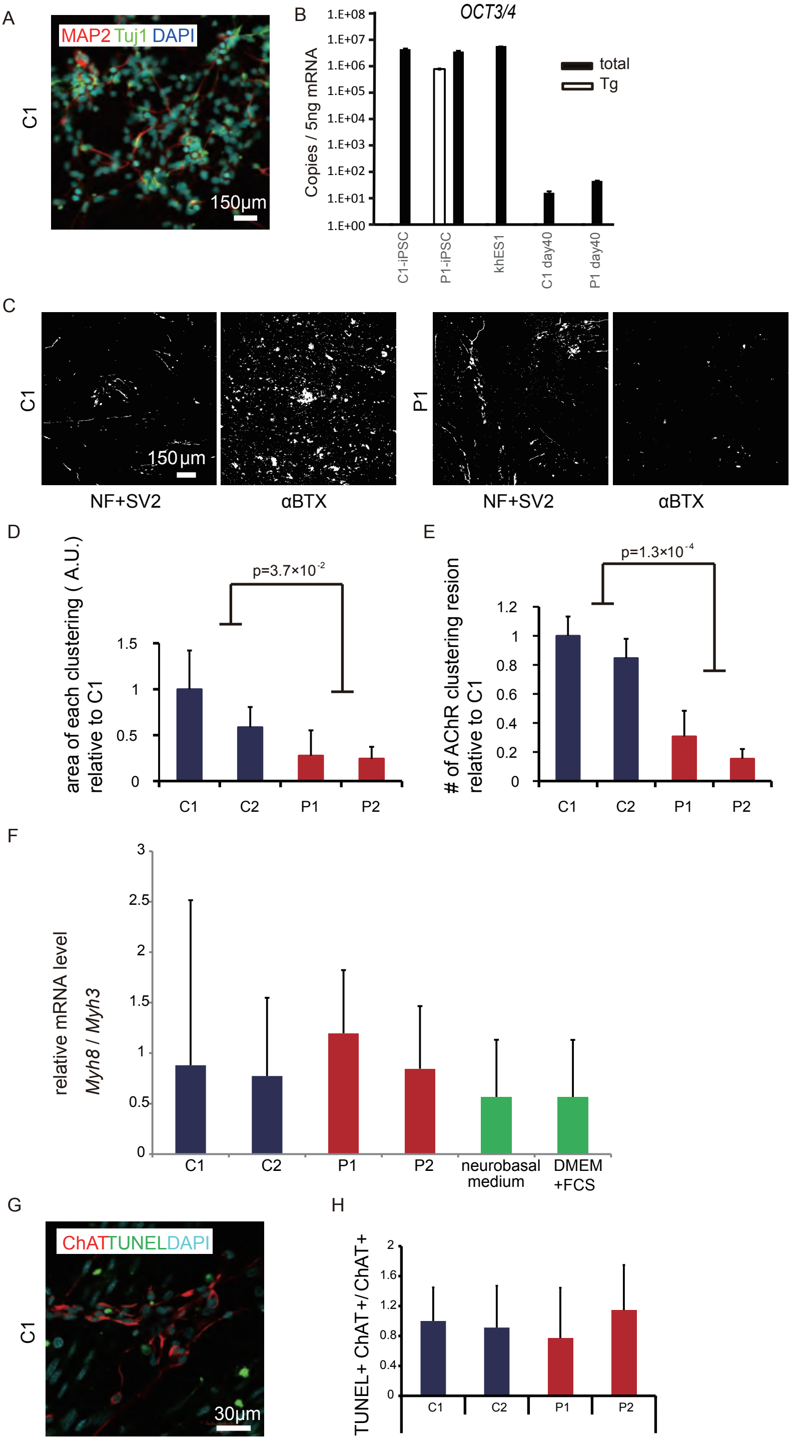
A
MAP2 Tuj1 DAPI
C1
150μm
B
OCT3/4
Copies / 5ng mRNA
total
Tg
C1-iPSC
P1-iPSC
khES1
C1 day40
P1 day40
C
C1
P1
150 μm
NF+SV2
αBTX
D
area of each clustering (A.U.)
relative to C1
p=3.7×10-2
E
# of AChR clustering resion
relative to C1
p=1.3×10-4
F
relative mRNA level
Myh8 / Myh3
C1
C2
P1
P2
neurobasal medium
DMEM +FCS
G
ChAT TUNEL DAPI
30μm
H
TUNEL+ ChAT+/ChAT+

Figure S3

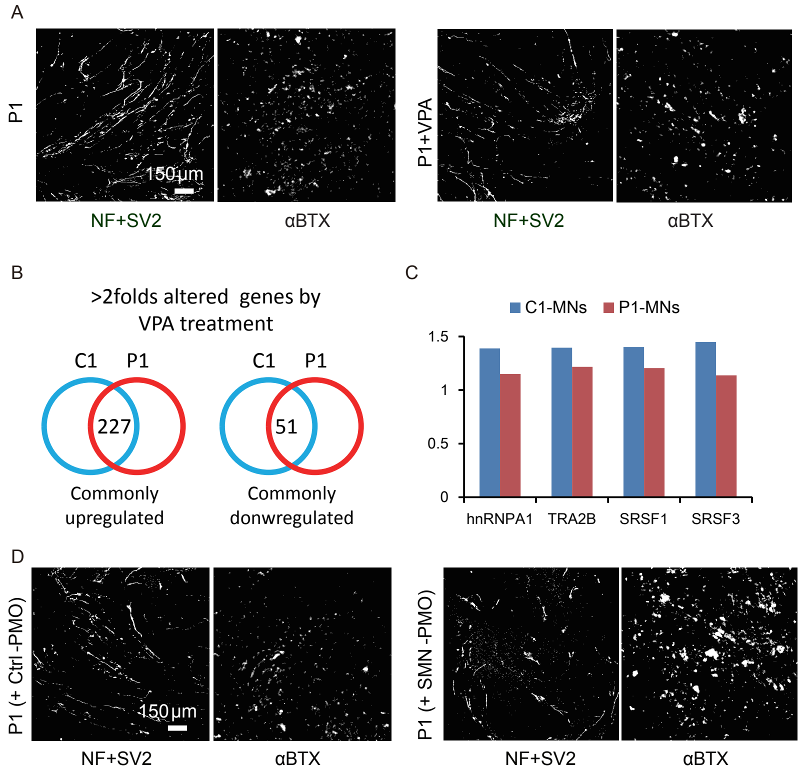
A
P1
150 μm
NF+SV2
αBTX
P1+VPA
NF+SV2
αBTX
B
>2folds altered genes by VPA treatment
C1
P1
227
Commonly upregulated
C1
P1
51
Commonly donwregulated
C
C1-MNs
P1-MNs
1.5
1
0.5
0
hnRNPA1
TRA2B
SRSF1
SRSF3
D
P1 (+ Ctrl-PMO)
150 μm
NF+SV2
αBTX
P1 (+ SMN-PMO)
NF+SV2
αBTX

Supplementary figure legends

Figure S1: Generation and characterization of SMA patient-specific iPSCs, related to Figure 1.
(A) Phase contrast images of SMA-iPSCs (P1, P2) showing typical pluripotent stem cell colony morphology. The immunocytochemical analysis revealed the expression of embryonic stem cell surface markers, SSEA-4, TRA-1-81. **(B)** The *NANOG* expression normalized to the *GAPDH* expression in control (C1, C2) and SMA-iPSCs (P1, P2) relative to hESCs (KhES1). Data are the means ±SEM of triplicate samples. **(C-E)** Microarray analysis of iPS clones. Control-fibroblasts are commercially available fibroblasts from a healthy volunteer. (C) Hierarchical clustering and (D) principal component analysis of iPSCs and parental fibroblasts. (E) Heatmap of gene sets preferentially expressed in pluripotent stem cells. **(F)** Methylation analysis of *OCT3/4* and *NANOG* promotor regions. Color boxes indicate methylated while white boxes indicate demethylated allele. **(G)** Karyotype analysis of iPSCs. **(H)** Teratoma formation assay showing successful differentiation of iPSCs into three germ layers. **(I)** The results of the quantitative RT-PCR analyses of *OCT3/4*, *SOX2* and *L-MYC* expression in control and SMA-iPSCs relative to hESCs (KhES1). ‘‘Tg’’ indicates primers detecting the transgene only, whereas ‘‘total’’ indicates primers detecting both the endogenous gene and the transgene. Scale bars: 100 μm. The data are the means ±SEM of triplicate samples.

Figure S2: Characteristics of iPSC-derived MNs and AChR clusters, related to Figure 2
(A) Immunostaining of SMA-iPSC-derived MNs. MAP2 (red) and Tuj1 (green) stained on day 40. **(B)** Quantitative RT-PCR analysis of the *OCT3/4* expression in the PSCs and PSC-derived MNs. The data are presented as the mean ±SEM of triplicate samples. **(C)** Processed black and white images used for automatic area counting of neurons and AChR clustering. NF+SV2 indicates neuron-positive areas and αBTX indicates AChR-clustering positive areas. **(D)** Quantitative analysis of the size of each AChR cluster. (means ±SEM, n=3, Wilcoxon rank-sum test). **(E)** Quantitative analysis of the number of AChR clusters. (means ±SEM, n=3, Wilcoxon rank-sum test). **(F)** Quantitative RT-PCR analysis of the expression of *Myh8* and *Myh3* genes in differentiated C2C12 myotubes. The ratio of *Myh8* to *Myh3* is shown. The data are presented as the mean ±SEM of triplicate dishes. Neurobasal medium; monocultured C2C12 cells were with co-culturing medium. DMEM+FCS; monocultured C2C12 cells with their maintenance medium. **(G)** The representative immunostaining image of iPSC-derived MNs with TUNEL (green) and ChAT (red) and **(H)** their quantification. The data are presented as the mean ±SEM of triplicate dishes.

Figure S3: Effect of VPA and PMO treatment on AChR clustering, related to Figure 3 and Figure 4.
(A) Processed black and white images used for automatic area counting of neurons and AChR clustering with or without VPA treatment. **(B)** Summary of RNA-seq analysis. **(C)** Expression levels of splicing factors (*SF2/ASF, Htra2-β1, SRp20, hnRNPA1*) with VPA treatment. Fold change to the expression levels without VPA treatment are shown. **(D)** Processed black and white images used for automatic area counting of neurons and AChR clustering after PMO treatment.

Table S1: list of genes upregulated by VPA treatment (>2 fold to compared to untreated), related to Figure 3.

TRIM34+TRIM6+TRIM6-TRIM34	CALCB	ARNTL
TMC6+TMC8	FGFR4	PPIC
ALS2CL	NCEH1	GSN
ETHE1	TMEM176B	SEMA3C
BLVRB	MITF	WNT16
FLJ46906	PIK3R5	EPOR
PPFIBP2	PPP1R1B	MFAP2
PTPN3	CACNA1G	SMYD3
NRGN	RTBDN	MUM1L1
PAQR5	RIT2	TAC3
AMBN	AKR1C3	TMEM255A
DUX2+DUX4+DUX4L2+DUX4L3+ DUX4L5+DUX4L6+DUX4L7	CXorf57	STEAP1
PAMR1	PENK	CRABP2
FSTL5	EGFL7	DENND2C
HOXD8	PLEKHB1	CAV2
DLEU1	GPR50	THEMIS2
CCDC152+SEPP1	NMNAT3	ALPL
CPT1A	ITM2A	TMCC3
MT2A	GLYATL1	GXYLT2
TMEM74	DPYD+DPYD-AS1	HLA-B
HLA-C	GABRE	ATP8B3
PRSS12	RAB7L1	LGALS1
CREG1	HLA-F+HLA-F-AS1	DNAJC12
SLC35F2	KCNJ4	IFI6
FXYD1	HEPH	IGSF1
DHRS2	ABCB1	CDK18
C4orf32	LRAT	TRAM1L1
NPAS1	VAV3	MIR1324
IL17RA	GPR158+GPR158-AS1	UTS2
PLCL1	SNORD114-8	GPC5
PLCG2	SERPINI1	PLEKHH2
DAPL1	CHRM4	IRF6
LPA	KCNIP2+LOC100289509	DPP10
NMRK1	CHRDL1	SULT1C4
TSPAN33	C9orf135	STAT4
USH2A	TESC	PAIP2B
IL16+STARD5	PRKCB	ARHGAP18
PPAP2C	KCTD19+PLEKHG4	C19orf77
TNNT1	TMEM176A	PRKCQ
ENPP5	GABRD	PPM1J
RASGRP2	CRYM	LPCAT2
SNORD61	HOXB7	PRKCG
HAPLN4	PDGFRA	C1orf115
FBLN2	ADRA1A	GRAMD1C
ARHGAP26	HPCA+TMEM54	TFEB
COL6A2	HERC5	ULBP1
DUSP23	KCNH3	ANO5
CHRNB4	OSTF1	TEX15
HLA-DPA1+HLA-DPB1	RAB11FIP1	GALNT10
SPAG6	MT1F	MIR5586
C3orf52	TPD52L1	RXRG
CYP2J2	SLC17A7	CD163L1
GALP	GMPR	C1QTNF9B+C1QTNF9B-AS1
STEAP1B	C4orf33	SLC2A4
SELL	CRLF1	TINCR
CRHR2	SNORA74A	LOC650368
CLDN6	TSPAN15	ALDH1A1
TMPRSS15	SCPEP1	SUSD3
IL13RA2	EDA	MIR3193
CD40	CLGN	C17orf96
RGS10	H2AFJ	DGAT2
MIR548AQ+MIR548AR	CD74	STAC2
C1QL1	MRAP2	SLC18A3
ITGA5	SYNPR	MIR7-3HG
GPX3	MIR4324	RNF128
NDST4	PLA2G4A	RLN2
PCBP3	ABCD2	FAM19A4
HLA-DMB	FTCD	RBM11
COL9A2	IFI30	C10orf128
TNNI3	A4GALT	LOC100506013
MICB	SLC9A9	RASD2
LOC401074	ARID5A	FAM101B
LOC401074	IMPA2	NRTN
MIR4735	MGARP	CHST9
SCGN	GPR37	ANKRD20A19P
ITIH5	SPINK2	

Table S2: list of genes downregulated by VPA treatment (>2 fold to compared to untreated), related to Figure 3.

EFCAB2	HYI+SZT2	MYD88
LINC00261	C4A+C4B+LOC100293534	LRFN4
TM4SF1	OBSL1	CLEC18A
BGLAP+PMF1+PMF1-BGLAP	GDPD2	SNORD4B
SNORD38B	ATP1A2	SNORD21
LPL	MIR93	MIR568
SNORD115-11+SNORD115-29+SNORD115-36+SNORD115-43	SERPINH1	ACSS1
LANCL2	SLC27A3	SNORD88A
AIFM2+H2AFY2	C4A+C4B+LOC100293534	SNORD12C
GLYCTK+MIR135A1	ACTG2	SOCS3
CLEC18C	MARVELD1	HIST1H2BC
MLC1	PDGFRB	MIR219-2+MIR2964A
SNORA51	RCBTB2	FADS2
MOV10	MIRLET7D	TAGLN
GTF2IRD2	MIR4508	MIR1305
MIR3175	MIR106B	CLEC18B
GTF2IRD2P1	SLC16A14	GTF2IRD2B

Table S3: primer sequences for PCR analysis, related to Figure 1-4.

primer name	Sequences (5' to 3')
NANOG	CCA AAG GCA AAC AAC CCA CTT
	GAC CGG GAC CTT GTC TTC CT
GAPDH	ACC ACA GTC CAT GCC ATC AC
	TCC ACC ACC CTG TTG CTG TA
total *OCT3/4*	CCC CAG GGC CCC ATT TTG GTA CC
	ACC TCA GTT TGA ATG CAT GGG AGA GC
transgene *OCT3/4*	CAT TCA AAC TGA GGT AAG GG
	TAG CGT AAA AGG AGC AAC ATA G
total *SOX2*	TTC ACA TGT CCC AGC ACT ACC AGA
	TCA CAT GTG TGA GAG GGG CAG TGT GC
transgene *SOX2*	TTC ACA TGT CCC AGC ACT ACC AGA
	TTT GTT TGA CAG GAG CGA CAA T
total *L-MYC*	GCG AAC CCA AGA CCC AGG CCT GCT CC
	CAG GGG GTC TGC TCG CAC CGT GAT G
transgene *L-MYC*	GGC TGA GAA GAG GAT GGC TAC
	TTT GTT TGA CAG GAG CGA CAA T
SMN-FL	CAAAAAGAAGGAAGGTGCTCACATT
	GTGTCATTTAGTGCTGCTCTATGC
SMN-⊿7	CTTGATGATGCTGATGCTTTGGGAAG
	CTATGCCAGCATTTCCATATAATAGCCAG
GAPDH	GTGGACCTGACCTGCCGTCT
	GGAGGAGTGGGTGTCGCTGT
Myh3	TGAGATTGCAGGATCTGGTGG
	CTCATGCTGGGCTTTCCTGA
Myh8	GCCGGGAGGTTCACACCAAA
	AAACCCAGAGAGGCAAGTGA

Supplementary experimental procedures

Study approval

Use of human ESCs was approved by the Ministry of Education, Culture, Sports, Science and Technology of Japan (MEXT). The study plan for recombinant DNA research has been approved by recombinant DNA experiments safety committee of Kyoto University. An experimental protocol was approved by the Animal Research Committee of Center for iPS cell research and application, Kyoto University.

Cell lines

The following fibroblasts were obtained from the NIGMS Human Genetic Cell Repository at the Coriell Institute for Medical Research: [GM00232, GM03813]. Donors of both fibroblasts were described as having SMA type I. The iPSCs generated from the GM00232 and GM03813 fibroblasts were renamed CiRA00070 and CiRA00069, respectively, according to the institutional regulations. A human embryonic stem cell line, KhES1, was kindly provided by Dr. Hirofumi Suemori (Institute for Frontier Medical Sciences, Kyoto University, Kyoto, Japan). The human iPS cell lines, 201B7 and 409B2, were kindly provided by Dr. Shinya Yamanaka (Center for iPS Cell Research and Application, Kyoto University, Kyoto, Japan). The murine myoblast cell line, C2C12, was kindly provided by Dr. Atsuko Sehara-Fujisawa (Institute for Frontier Medical Sciences, Kyoto University, Kyoto, Japan).

Establishment of iPSCs

Episomal vectors encoding reprogramming factors (*OCT3/4, SOX2, KLF4, L-MYC, LIN28* and p53 shRNA) were transduced into fibroblasts on day 0 as described previously (Okita et al., 2011). Plasmids were kindly provided by Dr. Keisuke Okita (CiRA, Kyoto University).The transfected cells were reseeded onto feeder layers on day 7, and maintained in embryonic stem cell medium (ReproCELL). Around day 30, the iPSC colonies were picked up as usual.

RNA isolation and quantitative PCR

Total RNA extraction from cells was performed using the RNeasy Mini kit (QIAGEN). One microgram of total RNA was used for reverse transcription with the PrimeScript RT Master Mix (TaKaRa). The real-time PCR was performed with SYBR Premix Ex TaqII (TaKaRa) in triplicate using the StepOnePlus system (Applied Biosystems). *GAPDH* was used as an endogenous control. The primer sets used for the quantitative PCR assay are described in **Table S3**.

Teratoma formation

The iPSCs recovered from one 6 cm dish were injected subcutaneously into NOG mice (Central Institute for Experimental Animals). Tumors were dissected eight weeks after injection and were fixed with PBS containing 4% paraformaldehyde. Paraffin-embedded tissues were sliced and stained with hematoxylin and eosin. Slides were examined using a BIOREVO BZ-9000 system (KEYENCE).

Genotyping (PCR-RFLP)

The methods used to detect homozygous *SMN1* exon 7 and 8 deletions were described previously (van der Steege et al., 1995). In brief, the restriction enzymes Dra I and Dde I cleave the PCR products originating only from *SMN2*, enabling *SMN1*-derived PCR products to be distinguished from those of *SMN2*. The completely

digested band corresponds to the deletion of exons 7 or 8 in *SMN1*. The results of the PCR electrophoresis were analyzed by a bioanalyzer (Agilent)(Burrell et al., 2011).

Protein isolation and Western blot analysis

Cells were isolated, suspended in M-PER (Thermo SCIENTIFIC), supplemented with 1% protease inhibitor cocktail (Sigma) and centrifuged at 20,000 g for 15 min at 4℃. A total of 3.125 μg of protein extracted from each sample was separated on 10% SDS-polyacrylamide gels, transferred to a nitrocellulose membrane, probed with a primary antibody against SMN (1:20,000; BD #610647), followed by horseradish-peroxidase-conjugated secondary antibody (1:10,000; Cell Signaling), and then visualized using ECL chemiluminescence reagents (Amersham). As a control, membranes were stripped and re-probed for β-actin (1:1,000; Cell Signaling #4970S). Quantitative densitometry analysis was performed using ImageQuant LAS 4000 software.

Immunocytochemistry and microscopy

Cells were fixed and permeabilized for 30 min at room temperature in 4% paraformaldehyde and 0.2% TritonX-100, and incubated with Block Ace (DS PHARMA BIOMEDICAL) to prevent any non-specific binding before overnight incubation with primary antibodies at 4℃. The following day, secondary antibody incubations were performed for 1 hour with the appropriate species-specific antiserum coupled to either FITC, Cy3 (Jackson ImmunoResearch, 1/100) or Alexa594 conjugated α-bungarotoxin (α-BTX) (Invitrogen #B13423, 1/500). After staining nuclei with DAPI (1/1,000), the cells were mounted utilizing Vectashield (Vector laboratories) and imaged using a LV1000 confocal microscope (Olympus). All antibodies were diluted in Block Ace. The following primary antibodies were used at the indicated concentrations: neurofilament 160 kDa (Millipore #MAB5254, 1/1000), Tuj1 (Covance #MMS435P, 1/2000), MAP2 (Sigma #M2320, 1/1000), HB9 (DSHB #81.5C10, 1/100), ChAT (Millipore #AB144P, 1/200), anti-synaptic vesicle protein 2 (DSHB #SV2, 1/20), NF-H (abcam #ab4680, 1/5000), mAb35 (DSHB #mAb35, 1:1,000), MHC (Millipore #A4.1025, 1:1,000) and TUNEL (Promega, DeadEnd Fluorometric TUNEL System).

Quantification of the results of the immunocytochemical analysis

Samples were imaged under identical gain and exposure settings. Only motor neurons containing a nucleus were counted in order to avoid double counting from adjoining sections. To calculate the average number of HB9-positive motor neurons, 12 visual fields in each preparation from each condition were counted using the BIOREVO BZ-9000 system (KEYENCE).

For the co-cultured samples, eight visual fields were evaluated and assessed in each preparation. Images were obtained with a 10×objective lens. For measurements, individual images of AChR clusters were imported into the ImageJ software program and split into red (endplate) and green (neurite and motor nerve terminal) channels. Each endplate and corresponding motor nerve terminal was automatically outlined and calculated using the IN Cell Analyzer 2000 software program (GE Healthcare Life Sciences), and areas greater than 427 pixels were kept as targets.

Microarray

Total RNA was extracted and purified using RNA mini kit (QIAGEN) according to manufacturer’s instruction. Gene expression analysis was performed using SurePrint G3 Human Gene Expression 8 × 60K kit (G4851A; Agilent technologies). Cy3-labelled cDNA was synthesized from 100 ng of RNA using Genomic DNA Enzymatic Labeling

Kit (Agilent Technologies). Labelled cDNA was fragmented with Gene Expression Hybridization Kit (Agilent Technologies), followed by hybridization at 65˚C for 17 hrs. Images were acquired by DNA Microarray Scanner (Agilent Technologies). The data were normalized by 75 percentile shift using the GeneSpring GX 12.6.1 software (Agilent Technologies). Principle component analysis (PCA) was performed by R ver 3.1.0. Two-way hierarchical clustering analysis was performed using Ward's method based on Euclidean distance. Heatmap was illustrated by GeneSpring GX 12.6.1 using average method based on Euclidean distance.

DNA methylation analysis

Genomic DNA was extracted and purified using PureLink Genomic DNA Mini Kit (Invitrogen) according to manufacturer's instruction. One μg of genomic DNA was subjected to bisulfite conversion of unmethylated cytosines of genomic DNA into uracils with EZ DNA Methylation Gold Kit (ZYMO Research). Promoter regions of *POU5F1* and *NANOG* were amplified by PCR from approximately 10 ng of bisulfite-treated DNA using Ex-taq (TaKaRa). PCR condition for *POU5F1* promoter was: 1 cycle of 98°C for 1 min, followed by 45 cycles of 98°C for 10 s, 60°C for 30 s, and 72°C for 1 min. That for *NANOG* promoter was: 1 cycle of 95°C for 3 min, followed by 40 cycles of 95°C for 30s, 58°C for 30 s, and 72°C for 1 min. Primers were: GTTTTTAGAGTAGTTGGGATTATAG and AACCCACCCTTATAAATTCTCAATTA for *NANOG*; ATTTGTTTTTTGGGTAGTTAAAGGT and CCAACTATCTTCATCTTAATAACATCC for *POU5F1*. Illumina libraries were generated by NEBNext Ultra DNA Library Prep Kit (New England BioLabs), and sequenced in 250 cycles Paired-end mode of MiSeq. All sequence reads were extracted in FASTQ format using MiSeq Reporter v2.3.32. Mapping to canonical sequence of human genome (hg19) was performed by bismark v.0.7.7 (Krueger and Andrews, 2011).

RNA-seq

After depletion of ribosomal RNA by RiboZero Gold (Epicentre), we prepared libraries using the Illumina TruSeq Stranded Total RNA Sample Prep kit. The libraries were sequenced in 100 cycle Single-Read mode of HiSeq2500. All sequence reads were extracted in FASTQ format using BCL2FASTQ Conversion Software 1.8.4 in the CASAVA 1.8.2 pipeline. The sequence reads were mapped to hg19 reference genes, downloaded on 10th December 2012, using Tophat v2.0.8b (Kim et al., 2013) and quantified by RPKMforGenes (Ramskold et al., 2009), downloaded on 19th, October 2012. Gene Ontology analysis was used by David v6.7 (Huang da et al., 2009).

GSE accession numbers

Microarray and RNA-seq deposited in GEO database can be accessed with the GEO accession number GSE65470 and GSE65508, respectively.

Motor neuron differentiation and co-culture with C2C12

The iPSCs were dissociated into single cells and quickly re-aggregated in DFK 5% medium (DMEM/F12 medium supplemented with KSR, NEAA, 2-mercaptoethanol, L-Glutamate, SB431542, dorsomorphin and Y27632) (9000 cells/150 μl/well) using 96-well low cell-adhesion plates (Lipidure-coat U96w from Nunc)(Eiraku et al., 2008; Morizane et al., 2011) . From day 8, the cell aggregates were treated with Sonic hedgehog (100 ng/ml) and retinoic acid (1 μM) for 1 week (Wada et al., 2009). On day 20, the cell aggregates were plated onto poly-l-lysin/laminin-coated culture dishes in neurobasal medium (Gibco) supplemented with the neurotrophic factors GDNF, BDNF and NT3 (10 ng/ml, R & D Systems). The medium was changed every 3-4 days thereafter.

For the co-culture with neuronal cells, the fusion of C2C12 myoblasts was induced by switching to the differentiation medium (DMEM supplemented with horse serum). On day 4, the MNs that had differentiated from the iPSCs (differentiation days 34-54) were harvested and plated on the induced myotubes, and the medium was changed to Neurobasal medium containing neurotrophic factors (BDNF, GDNF, NT3; 10 ng/ml each). Thereafter, the cultures were fed every two days by changing half of the medium.

VPA treatment

Co-cultured samples were treated with or without 1 mM VPA by changing half of the medium every two days. After six days of drug treatment, the area of NF and αBTX immunostaining was detected by immunocytochemistry and was analyzed by the IN Cell Analyzer 2000 software program.

PMO treatment

Designed PMOs SMN2E7D(-10-29) for suppressing splice silencing motifs in intron 7 of *SMN2* (Mitrpant et al., 2013) and its negative control were purchased from GENE TOOLS. SMN- or Ctrl-PMO (10 µM in medium) were introduced with the Endo-Porter (GENE TOOLS) on day 1 of co-culturing, and the cells were subsequently cultured for three days.

Statistics. Statistic functions in Microsoft Excel 2013 was used for statistical analyses. Results are expressed as mean ± SEM. Statistical significance was determined using Student's t test and Wilcoxon rank sum test. $P<0.05$ was considered significant. 'n' represents the number of independent experiments.

Supplemental References

Burrell, A., Foy, C., and Burns, M. (2011). Applicability of three alternative instruments for food authenticity analysis: GMO identification. Biotechnology research international *2011*, 838232.

Eiraku, M., Watanabe, K., Matsuo-Takasaki, M., Kawada, M., Yonemura, S., Matsumura, M., Wataya, T., Nishiyama, A., Muguruma, K., and Sasai, Y. (2008). Self-organized formation of polarized cortical tissues from ESCs and its active manipulation by extrinsic signals. Cell stem cell *3*, 519-532.

Huang da, W., Sherman, B.T., and Lempicki, R.A. (2009). Systematic and integrative analysis of large gene lists using DAVID bioinformatics resources. Nat Protoc *4*, 44-57.

Kim, D., Pertea, G., Trapnell, C., Pimentel, H., Kelley, R., and Salzberg, S.L. (2013). TopHat2: accurate alignment of transcriptomes in the presence of insertions, deletions and gene fusions. Genome Biol *14*, R36.

Krueger, F., and Andrews, S.R. (2011). Bismark: a flexible aligner and methylation caller for Bisulfite-Seq applications. Bioinformatics *27*, 1571-1572.

Mitrpant, C., Porensky, P., Zhou, H., Price, L., Muntoni, F., Fletcher, S., Wilton, S.D., and Burghes, A.H. (2013). Improved antisense oligonucleotide design to suppress aberrant SMN2 gene transcript processing: towards a treatment for spinal muscular atrophy. PLoS One *8*, e62114.

Morizane, A., Doi, D., Kikuchi, T., Nishimura, K., and Takahashi, J. (2011). Small-molecule inhibitors of bone morphogenic protein and activin/nodal signals promote highly efficient neural induction from human pluripotent stem cells. J Neurosci Res *89*, 117-126.

Okita, K., Matsumura, Y., Sato, Y., Okada, A., Morizane, A., Okamoto, S., Hong, H., Nakagawa, M., Tanabe, K., Tezuka, K.*, et al.* (2011). A more efficient method to generate integration-free human iPS cells. Nat Methods *8*, 409-412.

Ramskold, D., Wang, E.T., Burge, C.B., and Sandberg, R. (2009). An abundance of ubiquitously expressed genes revealed by tissue transcriptome sequence data. PLoS Comput Biol *5*, e1000598.

van der Steege, G., Grootscholten, P.M., van der Vlies, P., Draaijers, T.G., Osinga, J., Cobben, J.M., Scheffer, H., and Buys, C.H. (1995). PCR-based DNA test to confirm clinical diagnosis of autosomal recessive spinal muscular atrophy. Lancet *345*, 985-986.

Wada, T., Honda, M., Minami, I., Tooi, N., Amagai, Y., Nakatsuji, N., and Aiba, K. (2009). Highly efficient differentiation and enrichment of spinal motor neurons derived from human and monkey embryonic stem cells. PloS one *4*, e6722.
