# Supplementary material for: Modeling the Early Phenotype at the Neuromuscular Junction of Spinal Muscular Atrophy Using Patient-Derived iPSCs
Source: Stem Cell Reports. 2015 Mar 19;4(4):561–8. doi: 10.1016/j.stemcr.2015.02.010 (PMC4400613; doi:10.1016/j.stemcr.2015.02.010)
Supplement: Document S2. Article plus Supplemental Information [file mmc2.pdf]

Stem Cell Reports
Report

ISSCR
OPEN ACCESS

Modeling the Early Phenotype at the Neuromuscular Junction of Spinal Muscular Atrophy Using Patient-Derived iPSCs

Michiko Yoshida,[1,4] Shiho Kitaoka,[2,6] Naohiro Egawa,[2,5] Mayu Yamane,[1] Ryunosuke Ikeda,[1] Kayoko Tsukita,[2] Naoki Amano,[3] Akira Watanabe,[3] Masafumi Morimoto,[4] Jun Takahashi,[1] Hajime Hosoi,[4] Tatsutoshi Nakahata,[1] Haruhisa Inoue,[2,5] and Megumu K. Saito[1,*]

[1]Department of Clinical Application
[2]Department of Cell Growth and Differentiation
[3]Department of Reprogramming Science
Center for iPS Cell Research and Application (CiRA), Kyoto University, Kyoto 606-8507, Japan
[4]Department of Pediatrics, Graduate School of Medical Science, Kyoto Prefectural University of Medicine, Kyoto 602-8566, Japan
[5]Core Research for Evolutional Science and Technology (CREST), Japan Science and Technology Agency, Saitama 332-0012, Japan
[6]Present address: Division of Pharmacology, Department of Biochemistry and Molecular Biology, Kobe University Graduate School of Medicine, Kobe 650-0017, Japan
*Correspondence: msaito@cira.kyoto-u.ac.jp

http://dx.doi.org/10.1016/j.stemcr.2015.02.010

This is an open access article under the CC BY license (http://creativecommons.org/licenses/by/4.0/).

SUMMARY

Spinal muscular atrophy (SMA) is a neuromuscular disorder caused by mutations of the *survival of motor neuron 1* (*SMN1*) gene. In the pathogenesis of SMA, pathological changes of the neuromuscular junction (NMJ) precede the motor neuronal loss. Therefore, it is critical to evaluate the NMJ formed by SMA patients' motor neurons (MNs), and to identify drugs that can restore the normal condition. We generated NMJ-like structures using MNs derived from SMA patient-specific induced pluripotent stem cells (iPSCs), and found that the clustering of the acetylcholine receptor (AChR) is significantly impaired. Valproic acid and antisense oligonucleotide treatment ameliorated the AChR clustering defects, leading to an increase in the level of full-length SMN transcripts. Thus, the current in vitro model of AChR clustering using SMA patient-derived iPSCs is useful to dissect the pathophysiological mechanisms underlying the development of SMA, and to evaluate the efficacy of new therapeutic approaches.

INTRODUCTION

Proximal spinal muscular atrophy (SMA) is an autosomal recessive neuromuscular disorder caused by the homozygous deletion or mutation of the *survival of motor neuron 1* (*SMN1*) gene, resulting in a deficiency of the ubiquitously expressed SMN protein. Patients suffer from progressive muscular weakness, which eventually results in respiratory failure in severe case. Since there are no effective treatment options, SMA remains the most frequent genetic cause of infant mortality (Burghes and Beattie, 2009; Lefebvre et al., 1995). *SMN2*, a unique gene in humans, is an almost identical copy gene of *SMN1*, but has a constitutive C to T transition in its exon 7. This transition affects the splicing of *SMN2* mRNA, thereby resulting in the predominant production of a shorter unstable isoform termed SMN-Δ7 (Monani et al., 1999). Although *SMN2* is unable to compensate for the homozygous loss of *SMN1* because of the lower amount of full-length SMN transcripts (SMN-FL), the copy number of *SMN2* affects the severity of SMA (McAndrew et al., 1997).

Based on clinical examinations and the pathological analyses of end-stage specimens, SMA historically has been described as a lower motor neuron (MN) disease characterized by the degeneration of the anterior horn cells of the spinal cord, which subsequently leads to skeletal muscle atrophy and weakness (Dubowitz, 2009). However, recent studies in SMA animal models have shown that the earliest detectable pathological change is observed at the neuromuscular junctions (NMJs), including neurofilament (NF) accumulation at the endplate on postnatal day 1 (Ling et al., 2012). Subsequently, a central synaptic defect is observed on day 4, motor neuronal loss manifests around day 9, and almost all mice die by day 15 (Sleigh et al., 2011). Therefore, impairment of the NMJ structure appears to be one of the most important phenotypes, and the development of agents that target the NMJ pathology may represent an attractive approach for therapy. Indeed, an aberrant ultrastructure of NMJs also has been reported in a human prenatal specimen obtained from a fetus with type I SMA (Martínez-Hernández et al., 2013).

Despite recent advances in our understanding of the disease, the detailed mechanism(s) involved in the NMJ formation and maturation, which occur during both the prenatal and early postnatal periods, have not been fully described (Wu et al., 2012). With a few exceptions, the analyses of the pathological roles of SMN have been conducted mainly using animal models, because there are difficulties associated with obtaining human specimens from either biopsy or post-mortem samples. Although there are several available transgenic mouse models of SMA, inter-species differences between mice and humans, such as the

CrossMark

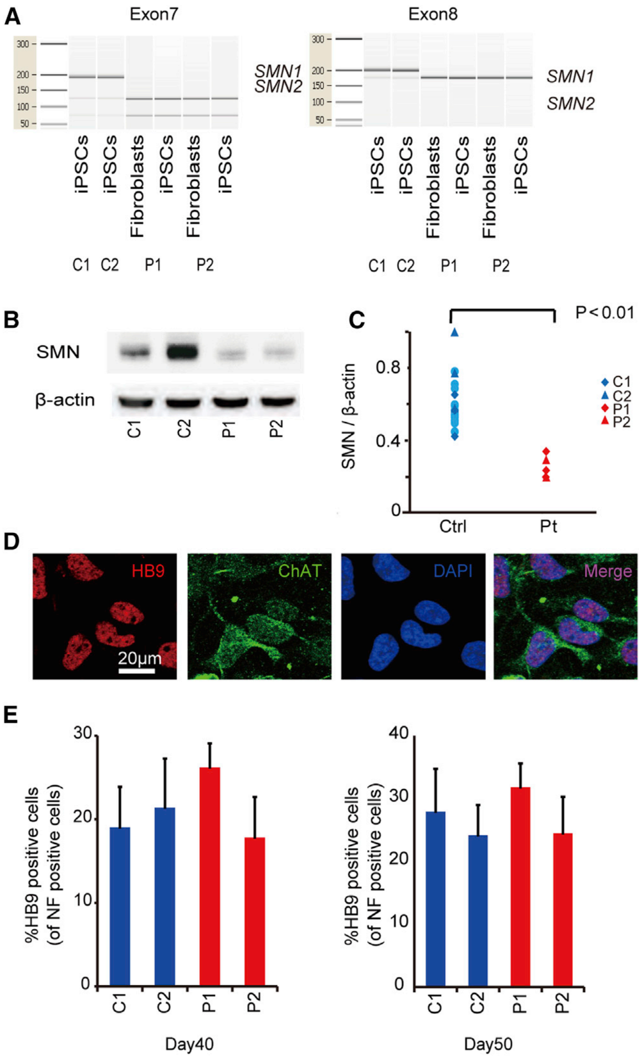

Figure 1. Differentiation of iPSCs into Spinal MNs

(A) The PCR restriction fragment-length polymorphism (RFLP) analysis using a bioanalyzer confirmed that the SMA-iPSCs maintained exon 7 and 8 deletions in the *SMN1* gene.

(B) Western blot analysis of SMN proteins.

(C) Quantification of the SMN protein expression relative to that of β-actin (eight control PSC clones and two SMA-iPSC clones) (n = 3, Wilcoxon rank-sum test).

(D) Immunostaining of SMA-iPSC-derived MNs. HB9, red; and ChAT, green on day 60.

(E) The quantitative immunocytochemical analysis for HB9-positive iPSC-derived MNs (means ± SEM, n = 3). See also Figure S1.

existence of *SMN2* in humans, hamper the translation of the findings in mouse studies to human clinical trials (Harahap et al., 2012; Martínez-Hernández et al., 2009; Park et al., 2010). Furthermore, there are difficulties related to evaluating the pathological roles of neurons and myocytes separately. To establish a platform to elucidate the pathology of the NMJ in SMA patients, we herein evaluated the ability of MNs from SMA patient-derived induced pluripotent stem cells (iPSCs) (Takahashi et al., 2007) to form NMJs.

RESULTS

Generation and Characterization of iPSCs from Type 1 SMA Patients

Fibroblasts from two independent type 1 SMA patients (Coriell IDs GM00232 and GM03813, referred to as P1 and P2) were reprogrammed by episomal vectors (Okita et al., 2011). Both SMA-iPSC clones used in this study (P1 and P2) showed a characteristic human embryonic stem cell (ESC)-like morphology, and expressed pluripotent markers compared to control ESC (KhES1) and iPSCs (201B7 and 409B2, referred to as C1 and C2) (Figures S1A and S1B). The RNA microarray analysis confirmed that the global gene expression pattern (Figures S1C and S1D) and levels of pluripotent stem cell-related genes (Figure S1E) in the P1 and P2 iPSCs were similar to that observed in the control iPSCs. The P1 and P2 iPSCs also exhibited demethylation of NANOG and OCT3/4 loci (Figure S1F) and maintained a normal karyotype (Figure S1G). Pluripotency of P1 and P2 iPSC lines were confirmed by teratoma formation assay (Figure S1H). The expression of introduced transgenes was rarely detected (Figure S1I). The genetic identity of the iPSC clones was proven by a short tandem repeat analysis (data not shown). SMA-iPSCs were confirmed to carry homozygosis deletions of exons 7 and 8 of the *SMN1* gene (Figure 1A; van der Steege et al., 1995), and their SMN protein level was also significantly lower than that in control iPSCs, including C1 and C2 (Figures 1B and 1C).

MN Differentiation of SMA-iPSCs

We next directed the SMA- and control iPSCs to differentiate into MNs using a previously reported cortical neuron (Morizane et al., 2011) and spinal MN differentiation protocol, with some modifications (Egawa et al., 2012). The iPSC-derived neurons expressed neuronal markers (Figure S2A) and MN-specific markers (Figure 1D). The expression of the introduced transgene *OCT3/4* detected in the P1-iPSCs was completely silenced after 40 days of MN differentiation (Figure S2B). Although a significant decline in MNs over time has been reported as a hallmark of SMA patient iPSC-derived MNs (Chang et al., 2011; Corti et al., 2012; Ebert et al., 2009), the two independent SMA-iPSC lines produced and maintained a similar number of HB9-positive MNs compared to control iPSCs after 40 and 50 days of differentiation (Figure 1E). Therefore, in our MN differentiation system, the SMA-iPSC lines were competent in generating mature MNs and presented no evidence of cell-autonomous MN loss by 50 days of differentiation.

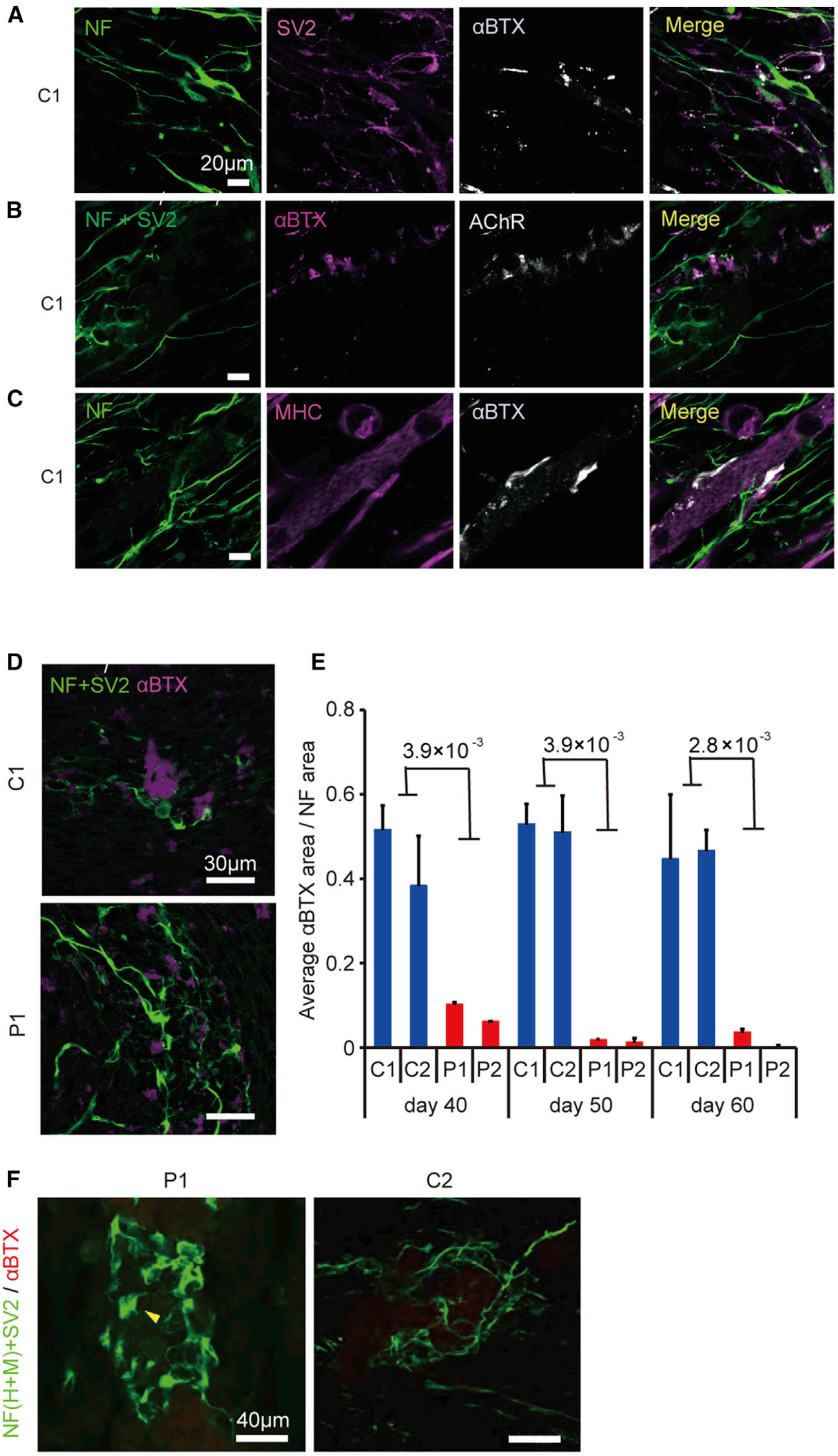

Figure 2. The Pre- and Post-synaptic Morphological Defects in Type 1 SMA

(A–D) Representative confocal micrographs showing the immunocytochemically labeled NMJ-LSs of iPSC-derived neurons and C2C12 myotubes. (A and B) Representative images of AChR clusters formed by C1 MNs. (C) AChR clusters stained with MHC. (D) NMJ-LSs of patient (P1)- and control (C1)-derived MNs on day 60.

(E) Quantitative immunocytochemical analysis of the α-BTX-positive area (means ± SEM, n = 3, Wilcoxon rank-sum test).

(F) Abnormal NF accumulation (yellow arrows) and poor terminal arborization of MNs in the SMA NMJ-LSs. See also Figure S2.

Formation of NMJ-like Structure with SMA-iPSCs

We next tried to develop an in vitro NMJ formation model using the human iPSC-derived MNs. We co-cultured control MNs with differentiated murine C2C12 cell lines and found that αBTX-positive AChRs were clustered at the site of SV2-positive neuronal endplates (Figure 2A). To exclude the presence of unexpected artifacts of αBTX staining under these conditions, we co-stained samples

with αBTX and anti-AChR antibodies and confirmed that the regions of both positive staining merged completely (Figure 2B). In addition, the AChR clusters were localized on myosin heavy chain (MHC)-positive multinuclear myotubes (Figure 2C).

We next evaluated the AChR clustering on myotubes co-cultured with SMA-iPSC-derived MNs and found it remarkably impaired (Figure 2D). We evaluated the area of αBTX to assess the ability of MNs to form and maintain the NMJ-like structures (NMJ-LSs). We evaluated the AChR clustering at several time points to determine whether MN maturation affects the phenotype of the NMJ-LSs. The SMA-iPSC-derived MNs induced far less AChR clustering in myotubes than control iPSC-derived MNs did at 40, 50, or 60 days of differentiation (Figures 2E and S2C). AChR clustering was rarely observed for either the SMA- or control iPSC-derived MNs at time points earlier than day 30 (data not shown). We also evaluated the average size of each AChR cluster (Figure S2D) and number of AChR clusters (Figure S2E). Consequently, the AChR clusters formed with SMA-iPSC-derived MNs were smaller and fewer in number than those formed with controls. To evaluate whether co-culturing with MNs affects the maturation status of C2C12, we compared the expression levels of embryonic (*Myh3*) and perinatal (*Myh8*) subtypes of MHCs in skeletal muscle with or without co-culturing (Stern-Straeter et al., 2011; Figure S2F). However, the ratio of expression of these genes was not different, indicating a lack of difference in the maturation of C2C12.

Although motor neuronal loss was not observed in our MN differentiation system without co-culture, there remains a possibility that the NMJ defects in SMA patient-derived MNs are due to MN death occurring under the co-culture conditions. To exclude the possibility, we performed TUNEL staining of the MNs (Figures S2G and S2H). The number of TUNEL-positive apoptotic MNs did not increase during co-culturing, which confirms that synapse loss indeed occurs in surviving MNs. The accumulation of NF proteins and poor arborization in distal axons and motor nerve terminals are considered to be specific features of SMA model mice, although their significance in the pathogenesis of human SMA is unknown (Cifuentes-Diaz et al., 2002; Kariya et al., 2008; Kong et al., 2009). These findings were observed in SMA-iPSC-derived MNs (Figure 2F), which indicates the functional deficit of the motor endplate in SMA. Taken together, the SMA-iPSC-derived MNs had impaired AChR clustering on myotubes in the absence of MN loss, indicating that there was functional impairment of MNs in terms of their forming or allowing for the maturation of NMJs.

The SMA Phenotype in NMJ-LS Was Rescued by Valproic Acid and Phosphorodiamidate Morpholino Oligonucleotides

Since the loss of NMJ formation is regarded to be an important hallmark preceding the motor neuronal loss, compounds that ameliorate the NMJ pathology may serve as promising therapeutic drug candidates. To evaluate whether the NMJ-LSs formation system used in our experiments can serve as a prototype for evaluating drug candidates, we assessed whether the SMN-inducing drug, valproic acid (VPA), could increase the AChR clustering in our co-culture system. VPA is known to increase the functional SMN protein by activating various promoters, including that of *SMN2*, and by correcting the abnormal splicing of *SMN2* exon 7, mainly through the upregulation of splicing factors (Harahap et al., 2012). Co-culturing myotubes and SMA-iPSC-derived MNs treated with VPA significantly increased the AChR clustering (Figures 3A–3C and S3A), while the clustering was not induced when monocultured myotubes were treated with VPA (data not shown). NF accumulation was not rescued by VPA treatment (Figure 3A). We confirmed that VPA treatment increased both the SMN-Δ7 and SMN-FL mRNA levels in the SMA-iPSC-derived MNs (Figure 3D).

We next performed an RNA sequencing (RNA-seq) analysis to evaluate the effects of VPA on the expression profiles of the MNs, and we found 227 genes that were upregulated more than 2-fold in the VPA-treated MNs, whereas 51 genes were downregulated (Figure S3B; Tables S1 and S2). The expression levels of splicing factors known to be affected by VPA treatment (Brichta et al., 2003; Harahap et al., 2012) were slightly upregulated as reported, in both the control and patient-derived MNs (Figure S3C).

To further explore the validity of NMJ-LSs, we next evaluated the effects of splicing modification on *SMN2*. Recently, the application of antisense oligonucleotides to promote *SMN2* exon 7 retention has been proposed as an alternative therapeutic approach for SMA (Mitrpant et al., 2013). For this purpose, we introduced phosphorodiamidate morpholino oligonucleotides (PMOs) targeting the intronic silencing motif in *SMN2* intron 7. Consequently, the SMN-specific PMO treatment dramatically improved AChR clustering with the patient-derived MNs (Figures 4A, 4B, and S3D). The PMO treatment also recovered the expression of SMN-FL (Figure 4C) and improved, at least partially, the abnormal NF accumulation (Figure 4A). We consider that these data indicate the potential therapeutic advantages of PMO for SMA patients. Overall, the NMJ-LS morphology could be useful for evaluating new therapeutic approaches for SMA.

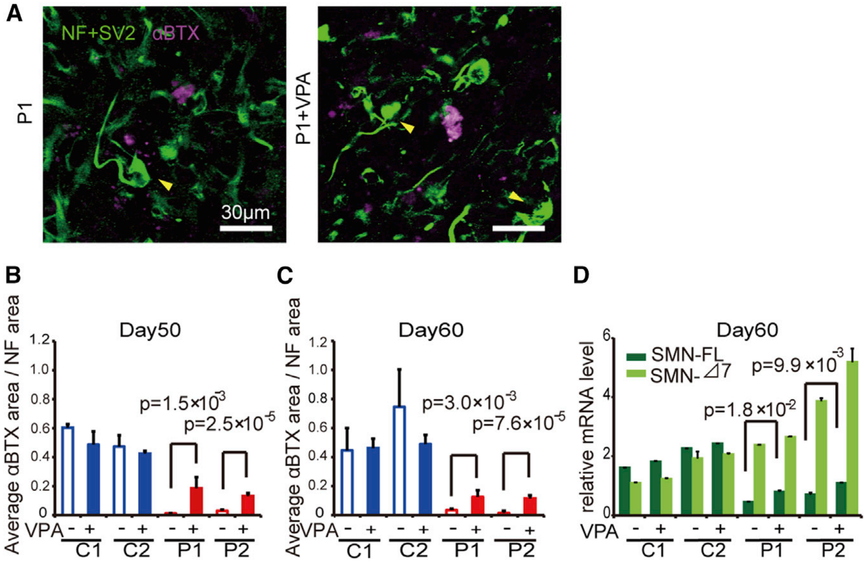

Figure 3. VPA Treatment Rescues the NMJ Pathology in SMA Patient-Derived Cell Cultures

(A) Representative images of NMJ-LSs formed with or without VPA. Yellow arrows indicate abnormal NF accumulation.

(B and C) Quantitative immunocytochemical analysis of the α-BTX area after VPA treatment (1 mM) (means ± SEM, n = 3, Student's t test).

(D) The mRNA levels of SMN-FL and SMN-Δ7 in SMA-iPSC MNs (means ± SEM, n = 3, Student's t test). See also Figure S3 and Tables S1 and S2.

DISCUSSION

Previous studies regarding the phenotype of MNs differentiated from SMA-iPSCs have focused mainly on the cell autonomous defects, such as shortened neurite extension, and the reduced size of the cell body; but, their significance in relation to the in vivo phenotype remains unclear (Chang et al., 2011; Ebert et al., 2009). Although motor neuronal loss during culture also has been reported, this is not observed in vivo until the end stage of the disease. We did not observe any progressive motor neuronal loss during culture, even during the longer time period, which is contrary to the previous reports (Chang et al., 2011; Corti et al., 2012; Ebert et al., 2009; Sareen et al., 2012). The precise reason for this difference is unknown, but a variety of methodological differences during culture, such as difference in the timing of the analysis, the protocol of MN differentiation, and the methods used for the evaluation, could all have contributed to this phenotypic variation. Although Corti et al. briefly reported the detection of NMJ defects after co-culturing human myoblasts and SMA-iPSC-derived MNs in a recent report (Corti et al., 2012), MN loss was observed without co-culturing the cells with myotubes in their SMA model, leaving the possibility

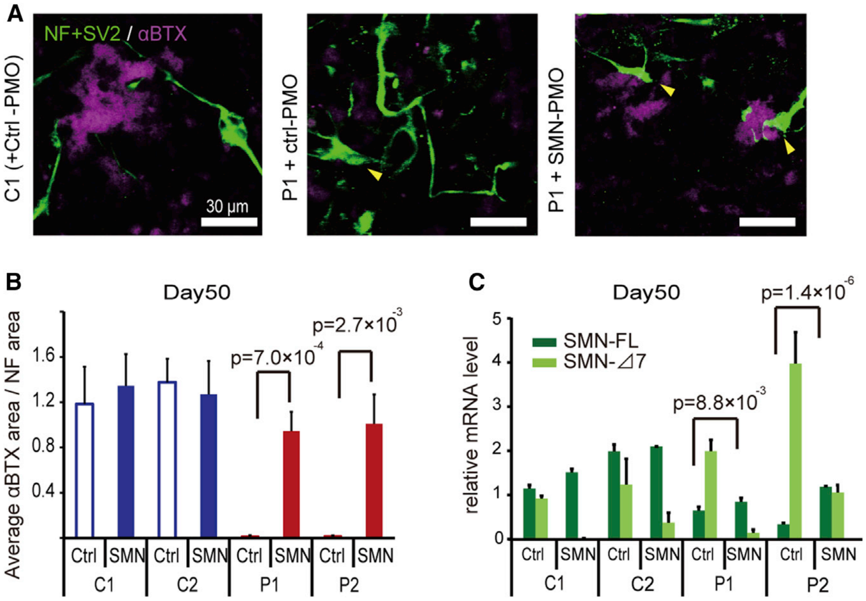

Figure 4. PMO Treatment Rescues the NMJ Pathology in SMA Patient-Derived Cell Cultures

(A) Representative images of NMJ-LSs formed with or without PMOs. Yellow arrows indicate abnormal NF accumulation.

(B) Quantitative immunocytochemical analysis of the α-BTX area after PMO treatment (means ± SEM, n = 3, Student's t test).

(C) The mRNA levels of SMN-FL and SMN-Δ7 with PMO treatment (means ± SEM, n = 3, Student's t test). See also Figure S3.

that preceding motor neuronal death may have led to the defect in forming the NMJ. In contrast, the patient-derived iPSCs used in our study yielded a similar ratio of HB9+ MNs compared to the two control lines. Moreover, we observed a significant reduction in AChR clustering, with no significant increases in the number of TUNEL-positive MNs, during co-culturing.

Since we focused on the pathology of NMJ, we performed a detailed examination of our NMJ-LS. To evaluate whether the developmental status of the MNs affects the NMJ pathology, we evaluated AChR clustering at three different time points and obtained consistent data. In addition to impaired AChR clustering, we detected abnormal presynaptic NF accumulation at the endplate in the SMA-iPSC-derived neurons, which also indicates that synaptic breakdown precedes motor neuronal death in our model. These data support a hypothesis that MNs derived from patient iPSCs are a major contributing factor to the pathogenesis in the NMJ due to SMA. Based on these observations, we considered that the morphological defect of NMJ-LS in our culture was due to functional impairments of the MNs in target pathfinding and/or in inducing or maintaining AChR clustering, rather than due to motor neuronal loss. Considering that the formation and maintenance of NMJs has been indicated to precede the occurrence of MN death even in humans, as mentioned above, the vulnerability of MNs in SMA patients seems to be due not only to the autonomous cell susceptibility to various stresses, but also as a consequence of the NMJ defect, which causes the impairment of neurotrophic factors and subsequent death of MNs (Fidziańska and Rafalowska, 2002; Fischer et al., 2004).

In summary, we demonstrated that the early SMA phenotype in NMJ could be recapitulated with MN differentiated from SMA-iPSCs. Since the available outcome measures to assess the drug efficacy in SMA are limited, our findings that the NMJ is a vulnerable target amenable to rescue by VPA and PMOs seems to indicate that this system will be useful for future evaluations of novel therapeutic candidates. Further experiments with patient-derived iPSCs on the neurodevelopmental aspects of the neuromuscular system, including specific molecular and cellular functions of SMN in both muscle and MN, will provide new insights into the pathophysiology of SMA. We believe that this approach will also help deepen our understanding of the pathogenesis of the muscle and MN interactions on the formation of the NMJ.

EXPERIMENTAL PROCEDURES

Study Approval

Use of human ESCs was approved by the Ministry of Education, Culture, Sports, Science and Technology of Japan (MEXT). The study plan for recombinant DNA research has been approved by the recombinant DNA experiments safety committee of Kyoto University. An experimental protocol was approved by the Animal Research Committee of CiRA, Kyoto University.

MN Differentiation and Co-culture with C2C12

The iPSCs were dissociated into single cells and quickly re-aggregated in DFK 5% medium (DMEM/F12 medium supplemented with KSR, NEAA, 2-mercaptoethanol, L-Glutamate, SB431542, dorsomorphin, and Y27632) (9,000 cells/150 μl/well) using 96-well low cell-adhesion plates (Lipidure-coat U96w from Nunc) (Eiraku et al., 2008; Morizane et al., 2011). From day 8, the cell aggregates were treated with Sonic hedgehog (100 ng/ml) and retinoic acid (1 μM) for 1 week (Wada et al., 2009). On day 20, the cell aggregates were plated onto poly-l-lysin/laminin-coated culture dishes in neuronal medium (neurobasal medium [Gibco] supplemented with the neurotrophic factors GDNF, BDNF, and NT3 [10 ng/ml, R&D Systems]). The medium was changed every 3 to 4 days thereafter.

For the co-culture with neuronal cells, the fusion of C2C12 myoblasts was induced by switching to the differentiation medium (DMEM supplemented with horse serum). On day 4, the MNs that had differentiated from the iPSCs (differentiation days 34–54) were harvested and plated on the induced myotubes, and the medium was changed to neuronal medium. Thereafter, the cultures were fed every 2 days by changing half of the medium.

VPA Treatment

Co-cultured samples were treated with or without 1 mM VPA by changing half of the medium every 2 days. After 6 days of drug treatment, the area of NF and αBTX immunostaining was detected by immunocytochemistry and was analyzed by the IN Cell Analyzer 2000 software program.

PMO Treatment

Designed PMOs SMN2E7D(-10-29) for suppressing splice silencing motifs in intron 7 of *SMN2* (Mitrpant et al., 2013) and its negative control were purchased from Gene Tools. SMN- or Ctrl-PMO (10 μM in medium) were introduced with the Endo-Porter (Gene Tools) on day 1 of co-culturing, and the cells were subsequently cultured for 3 days.

Statistics

Statistic functions in Microsoft Excel 2013 were used for statistical analyses. Statistical significance was determined using Student's t test and Wilcoxon rank-sum test, $p < 0.05$ was considered significant, and n represents the number of independent experiments.

SUPPLEMENTAL INFORMATION

Supplemental Information includes Supplemental Experimental Procedures, three figures, and three tables and can be found with this article online at http://dx.doi.org/10.1016/j.stemcr.2015.02.010.

AUTHOR CONTRIBUTIONS

M.Yo., S.K., M.M., H.H., J.T., T.N., H.I., and M.K.S. designed the research. M.Yo., S.K., N.E., M.Ya., R.I., N.A., and K.T. performed the research. M.Yo., A.W., M.Ya., and R.I. analyzed the data. M.Yo. and M.K.S. wrote the paper.

ACKNOWLEDGMENTS

We are grateful to Y. Sasaki, Y. Jindai, S. Nakamura, S. Benno, and T. Ohkame for their technical assistance. We also thank A. Niwa, K. Oshima, T. Tanaka, and K. Chiyonobu for scientific comments, and H. Watanabe for administrative assistance. We are grateful to Dr. Keisuke Okita for plasmid distribution and scientific comments. This work was supported by grants from Ministry of Health, Labour and Welfare of Japan; grants from the Ministry of Education, Culture, Sports, Science and Technology of Japan; the Leading Project of Ministry of Education, Culture, Sports, Science and Technology (T.N.); the Funding Program for World-Leading Innovative Research and Development on Science and Technology of the Japan Society for the Promotion of Science (T.N. and M.K.S.); the grant for Core Center for iPS Cell Research of Research Center Network for Realization of Regenerative Medicine from the Japan Science and Technology Agency (JST) (T.N., H.I, and M.K.S.); CREST (H.I.); the Ministry of Health, Labour and Welfare of Japan (H.I.); the Ministry of Education, Culture, Sports, Science and Technology of Japan (Innovative Area Foundation of Synapse and Neurocircuit Pathology [22110007] to H.I.); the Program for Intractable Diseases Research utilizing disease-specific iPS cells of JST (H.I. and T.N.); the Japan Research Foundation for Clinical Pharmacology (H.I.); the Mochida Memorial Foundation for Medical and Pharmaceutical Research (H.I.); and Intramural Research Grant (24-9) for Neurological and Psychiatry Disorders of NCNP (H.I.).

Received: October 4, 2013
Revised: February 12, 2015
Accepted: February 12, 2015
Published: March 19, 2015

REFERENCES

Brichta, L., Hofmann, Y., Hahnen, E., Siebzehnrubl, F.A., Raschke, H., Blumcke, I., Eyupoglu, I.Y., and Wirth, B. (2003). Valproic acid increases the SMN2 protein level: a well-known drug as a potential therapy for spinal muscular atrophy. Hum. Mol. Genet. *12*, 2481–2489.

Burghes, A.H., and Beattie, C.E. (2009). Spinal muscular atrophy: why do low levels of survival motor neuron protein make motor neurons sick? Nat. Rev. Neurosci. *10*, 597–609.

Chang, T., Zheng, W., Tsark, W., Bates, S., Huang, H., Lin, R.J., and Yee, J.K. (2011). Brief report: phenotypic rescue of induced pluripotent stem cell-derived motoneurons of a spinal muscular atrophy patient. Stem Cells *29*, 2090–2093.

Cifuentes-Diaz, C., Nicole, S., Velasco, M.E., Borra-Cebrian, C., Panozzo, C., Frugier, T., Millet, G., Roblot, N., Joshi, V., and Melki, J. (2002). Neurofilament accumulation at the motor endplate and lack of axonal sprouting in a spinal muscular atrophy mouse model. Hum. Mol. Genet. *11*, 1439–1447.

Corti, S., Nizzardo, M., Simone, C., Falcone, M., Nardini, M., Ronchi, D., Donadoni, C., Salani, S., Riboldi, G., Magri, F., et al. (2012). Genetic correction of human induced pluripotent stem cells from patients with spinal muscular atrophy. Sci. Transl. Med. *4*, 165ra162.

Dubowitz, V. (2009). Ramblings in the history of spinal muscular atrophy. Neuromuscul. Disord. *19*, 69–73.

Ebert, A.D., Yu, J., Rose, F.F., Jr., Mattis, V.B., Lorson, C.L., Thomson, J.A., and Svendsen, C.N. (2009). Induced pluripotent stem cells from a spinal muscular atrophy patient. Nature *457*, 277–280.

Egawa, N., Kitaoka, S., Tsukita, K., Naitoh, M., Takahashi, K., Yamamoto, T., Adachi, F., Kondo, T., Okita, K., Asaka, I., et al. (2012). Drug screening for ALS using patient-specific induced pluripotent stem cells. Sci. Transl. Med. *4*, 145ra104.

Eiraku, M., Watanabe, K., Matsuo-Takasaki, M., Kawada, M., Yonemura, S., Matsumura, M., Wataya, T., Nishiyama, A., Muguruma, K., and Sasai, Y. (2008). Self-organized formation of polarized cortical tissues from ESCs and its active manipulation by extrinsic signals. Cell Stem Cell *3*, 519–532.

Fidziańska, A., and Rafalowska, J. (2002). Motoneuron death in normal and spinal muscular atrophy-affected human fetuses. Acta Neuropathol. *104*, 363–368.

Fischer, L.R., Culver, D.G., Tennant, P., Davis, A.A., Wang, M., Castellano-Sanchez, A., Khan, J., Polak, M.A., and Glass, J.D. (2004). Amyotrophic lateral sclerosis is a distal axonopathy: evidence in mice and man. Exp. Neurol. *185*, 232–240.

Harahap, I.S., Saito, T., San, L.P., Sasaki, N., Gunadi, Nurputra, D.K., Yusoff, S., Yamamoto, T., Morikawa, S., Nishimura, N., et al. (2012). Valproic acid increases SMN2 expression and modulates SF2/ASF and hnRNPA1 expression in SMA fibroblast cell lines. Brain Dev. *34*, 213–222.

Kariya, S., Park, G.H., Maeno-Hikichi, Y., Leykekhman, O., Lutz, C., Arkovitz, M.S., Landmesser, L.T., and Monani, U.R. (2008). Reduced SMN protein impairs maturation of the neuromuscular junctions in mouse models of spinal muscular atrophy. Hum. Mol. Genet. *17*, 2552–2569.

Kong, L., Wang, X., Choe, D.W., Polley, M., Burnett, B.G., Bosch-Marcé, M., Griffin, J.W., Rich, M.M., and Sumner, C.J. (2009). Impaired synaptic vesicle release and immaturity of neuromuscular junctions in spinal muscular atrophy mice. J. Neurosci. *29*, 842–851.

Lefebvre, S., Bürglen, L., Reboullet, S., Clermont, O., Burlet, P., Viollet, L., Benichou, B., Cruaud, C., Millasseau, P., Zeviani, M., et al. (1995). Identification and characterization of a spinal muscular atrophy-determining gene. Cell *80*, 155–165.

Ling, K.K., Gibbs, R.M., Feng, Z., and Ko, C.P. (2012). Severe neuromuscular denervation of clinically relevant muscles in a mouse model of spinal muscular atrophy. Hum. Mol. Genet. *21*, 185–195.

Martínez-Hernández, R., Soler-Botija, C., Also, E., Alías, L., Caselles, L., Gich, I., Bernal, S., and Tizzano, E.F. (2009). The developmental pattern of myotubes in spinal muscular atrophy indicates prenatal delay of muscle maturation. J. Neuropathol. Exp. Neurol. *68*, 474–481.

Martínez-Hernández, R., Bernal, S., Also-Rallo, E., Alías, L., Barceló, M.J., Hereu, M., Esquerda, J.E., and Tizzano, E.F. (2013). Synaptic defects in type I spinal muscular atrophy in human development. J. Pathol. *229*, 49–61.

McAndrew, P.E., Parsons, D.W., Simard, L.R., Rochette, C., Ray, P.N., Mendell, J.R., Prior, T.W., and Burghes, A.H. (1997). Identification of proximal spinal muscular atrophy carriers and patients by analysis of SMNT and SMNC gene copy number. Am. J. Hum. Genet. *60*, 1411–1422.

Mitrpant, C., Porensky, P., Zhou, H., Price, L., Muntoni, F., Fletcher, S., Wilton, S.D., and Burghes, A.H. (2013). Improved antisense oligonucleotide design to suppress aberrant SMN2 gene transcript processing: towards a treatment for spinal muscular atrophy. PLoS ONE *8*, e62114.

Monani, U.R., Lorson, C.L., Parsons, D.W., Prior, T.W., Androphy, E.J., Burghes, A.H., and McPherson, J.D. (1999). A single nucleotide difference that alters splicing patterns distinguishes the SMA gene SMN1 from the copy gene SMN2. Hum. Mol. Genet. *8*, 1177–1183.

Morizane, A., Doi, D., Kikuchi, T., Nishimura, K., and Takahashi, J. (2011). Small-molecule inhibitors of bone morphogenic protein and activin/nodal signals promote highly efficient neural induction from human pluripotent stem cells. J. Neurosci. Res. *89*, 117–126.

Okita, K., Matsumura, Y., Sato, Y., Okada, A., Morizane, A., Okamoto, S., Hong, H., Nakagawa, M., Tanabe, K., Tezuka, K., et al. (2011). A more efficient method to generate integration-free human iPS cells. Nat. Methods *8*, 409–412.

Park, G.H., Maeno-Hikichi, Y., Awano, T., Landmesser, L.T., and Monani, U.R. (2010). Reduced survival of motor neuron (SMN) protein in motor neuronal progenitors functions cell autonomously to cause spinal muscular atrophy in model mice expressing the human centromeric (SMN2) gene. J. Neurosci. *30*, 12005–12019.

Sareen, D., Ebert, A.D., Heins, B.M., McGivern, J.V., Ornelas, L., and Svendsen, C.N. (2012). Inhibition of apoptosis blocks human motor neuron cell death in a stem cell model of spinal muscular atrophy. PLoS ONE *7*, e39113.

Sleigh, J.N., Gillingwater, T.H., and Talbot, K. (2011). The contribution of mouse models to understanding the pathogenesis of spinal muscular atrophy. Dis. Model. Mech. *4*, 457–467.

Stern-Straeter, J., Bonaterra, G.A., Kassner, S.S., Zügel, S., Hörmann, K., Kinscherf, R., and Goessler, U.R. (2011). Characterization of human myoblast differentiation for tissue-engineering purposes by quantitative gene expression analysis. J. Tissue Eng. Regen. Med. *5*, e197–e206.

Takahashi, K., Tanabe, K., Ohnuki, M., Narita, M., Ichisaka, T., Tomoda, K., and Yamanaka, S. (2007). Induction of pluripotent stem cells from adult human fibroblasts by defined factors. Cell *131*, 861–872.

van der Steege, G., Grootscholten, P.M., van der Vlies, P., Draaijers, T.G., Osinga, J., Cobben, J.M., Scheffer, H., and Buys, C.H. (1995). PCR-based DNA test to confirm clinical diagnosis of autosomal recessive spinal muscular atrophy. Lancet *345*, 985–986.

Wada, T., Honda, M., Minami, I., Tooi, N., Amagai, Y., Nakatsuji, N., and Aiba, K. (2009). Highly efficient differentiation and enrichment of spinal motor neurons derived from human and monkey embryonic stem cells. PLoS ONE *4*, e6722.

Wu, H., Lu, Y., Shen, C., Patel, N., Gan, L., Xiong, W.C., and Mei, L. (2012). Distinct roles of muscle and motoneuron LRP4 in neuromuscular junction formation. Neuron *75*, 94–107.

Stem Cell Reports, Volume *4*

Supplemental Information

Modeling the Early Phenotype at the Neuromuscular Junction of Spinal Muscular Atrophy Using Patient-Derived iPSCs

Michiko Yoshida, Shiho Kitaoka, Naohiro Egawa, Mayu Yamane, Ryunosuke Ikeda, Kayoko Tsukita, Naoki Amano, Akira Watanabe, Masafumi Morimoto, Jun Takahashi, Hajime Hosoi, Tatsutoshi Nakahata, Haruhisa Inoue, and Megumu K. Saito

Figure S1

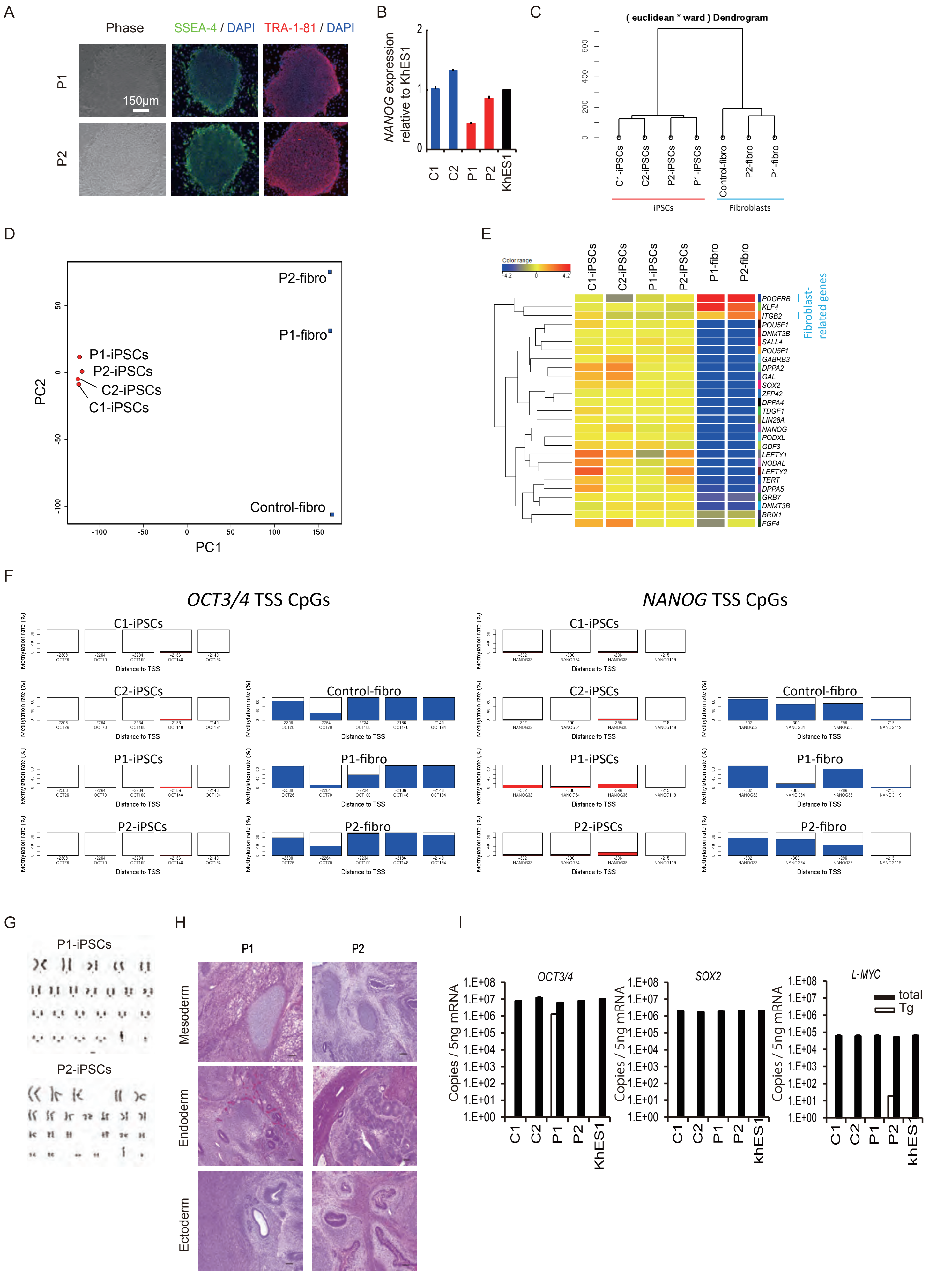

A
Phase
SSEA-4 / DAPI
TRA-1-81 / DAPI
P1
P2
150μm
B
NANOG expression relative to KhES1
C1
C2
P1
P2
KhES1
C
(euclidean * ward) Dendrogram
C1-iPSCs
C2-iPSCs
P2-iPSCs
P1-iPSCs
Control-fibro
P2-fibro
P1-fibro
iPSCs
Fibroblasts
D
P2-fibro
P1-fibro
P1-iPSCs
P2-iPSCs
C2-iPSCs
C1-iPSCs
Control-fibro
PC1
PC2
E
Color range
C1-iPSCs
C2-iPSCs
P1-iPSCs
P2-iPSCs
P1-fibro
P2-fibro
PDGFRB
KLF4
ITGB2
POU5F1
DNMT3B
SALL4
POU5F1
GABRB3
DPPA2
GAL
SOX2
ZFP42
DPPA4
TDGF1
LIN28A
NANOG
PODXL
GDF3
LEFTY1
NODAL
LEFTY2
TERT
DPPA5
GRB7
DNMT3B
BRIX1
FGF4
Fibroblast-related genes
F
OCT3/4 TSS CpGs
NANOG TSS CpGs
C1-iPSCs
C2-iPSCs
P1-iPSCs
P2-iPSCs
Control-fibro
P1-fibro
P2-fibro
Methylation rate (%)
Distance to TSS
G
P1-iPSCs
P2-iPSCs
H
P1
P2
Mesoderm
Endoderm
Ectoderm
I
OCT3/4
SOX2
L-MYC
Copies / 5ng mRNA
total
Tg
C1
C2
P1
P2
KhES1

Figure S2

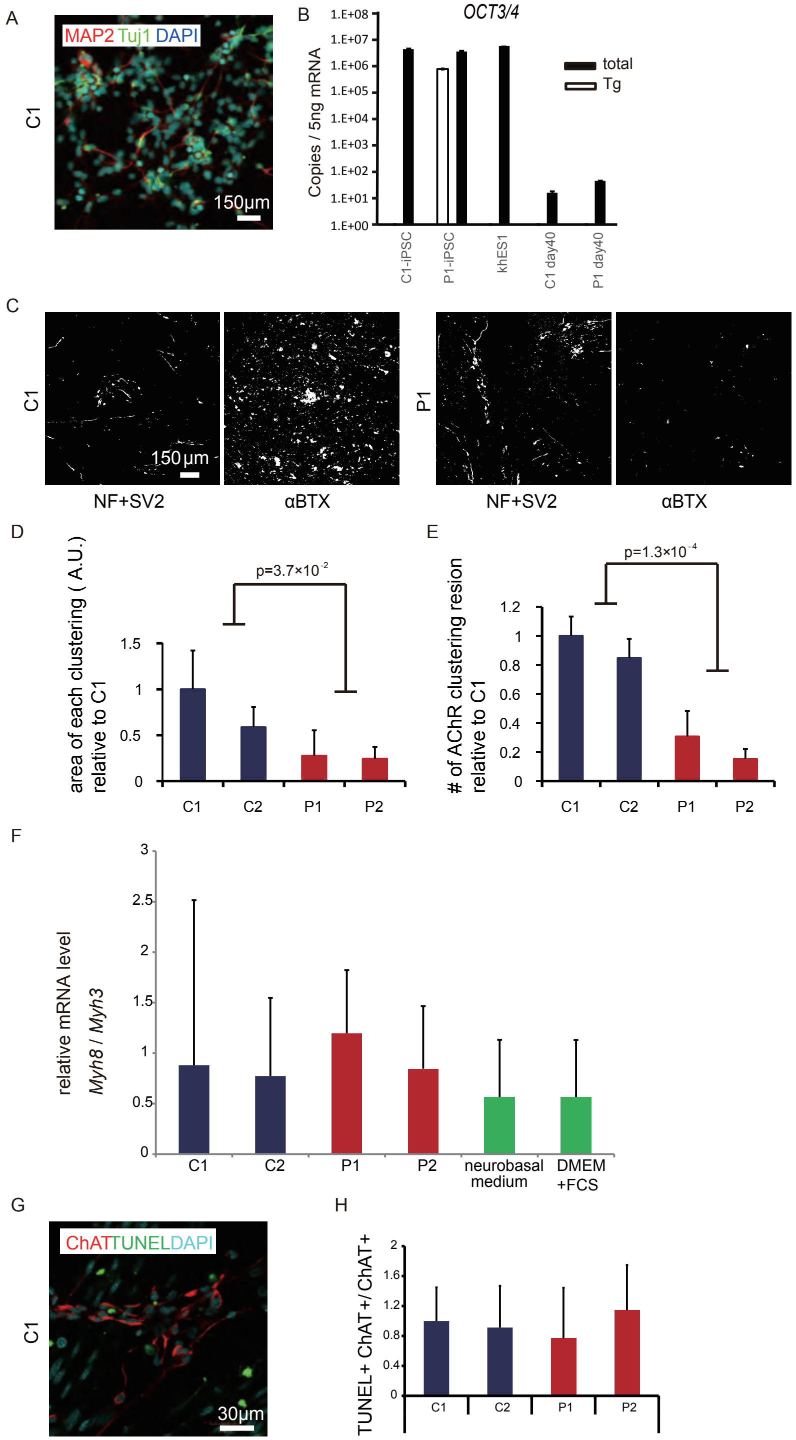
A
C1
MAP2 Tuj1 DAPI
150μm
B
OCT3/4
Copies / 5ng mRNA
1.E+08
1.E+07
1.E+06
1.E+05
1.E+04
1.E+03
1.E+02
1.E+01
1.E+00
total
Tg
C1-iPSC
P1-iPSC
khES1
C1 day40
P1 day40
C
C1
150 μm
NF+SV2
αBTX
P1
NF+SV2
αBTX
D
area of each clustering (A.U.)
relative to C1
p=3.7×10-2
1.5
1
0.5
0
C1
C2
P1
P2
E
# of AChR clustering resion
relative to C1
p=1.3×10-4
1.2
1
0.8
0.6
0.4
0.2
0
C1
C2
P1
P2
F
relative mRNA level
Myh8 / Myh3
3
2.5
2
1.5
1
0.5
0
C1
C2
P1
P2
neurobasal medium
DMEM +FCS
G
C1
ChATTUNELDAPI
30μm
H
TUNEL+ ChAT+/ChAT+
2
1.6
1.2
0.8
0.4
0
C1
C2
P1
P2

Figure S3

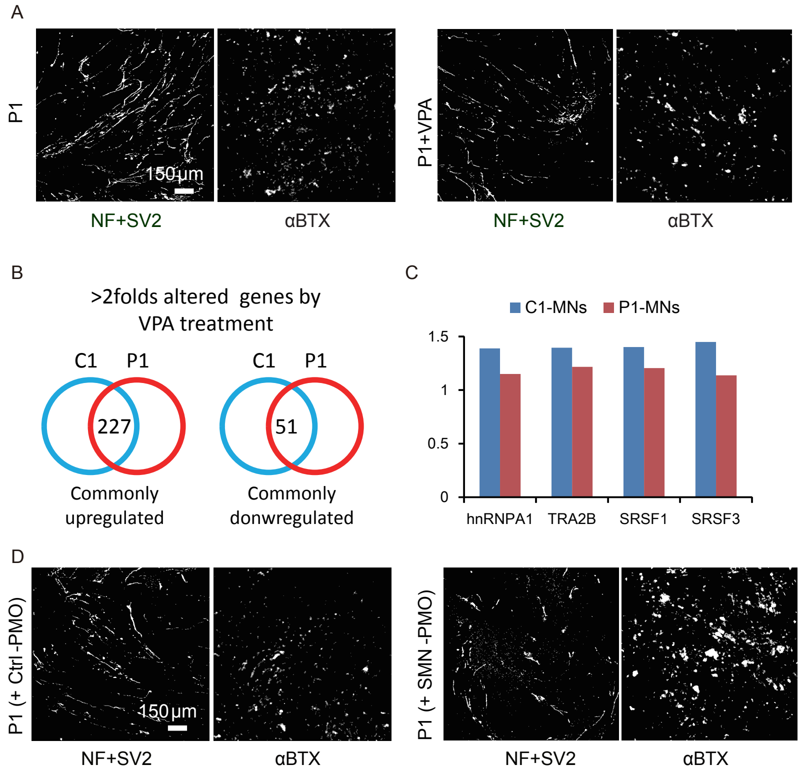
A
P1
150 µm
NF+SV2
αBTX
P1+VPA
NF+SV2
αBTX
B
>2folds altered genes by
VPA treatment
C1
P1
227
Commonly
upregulated
C1
P1
51
Commonly
donwregulated
C
C1-MNs
P1-MNs
1.5
1
0.5
0
hnRNPA1
TRA2B
SRSF1
SRSF3
D
P1 (+ Ctrl-PMO)
150 µm
NF+SV2
αBTX
P1 (+ SMN-PMO)
NF+SV2
αBTX

Supplementary figure legends

Figure S1: Generation and characterization of SMA patient-specific iPSCs, related to Figure 1.

(A) Phase contrast images of SMA-iPSCs (P1, P2) showing typical pluripotent stem cell colony morphology. The immunocytochemical analysis revealed the expression of embryonic stem cell surface markers, SSEA-4, TRA-1-81. **(B)** The *NANOG* expression normalized to the *GAPDH* expression in control (C1, C2) and SMA-iPSCs (P1, P2) relative to hESCs (KhES1). Data are the means ±SEM of triplicate samples. **(C-E)** Microarray analysis of iPS clones. Control-fibroblasts are commercially available fibroblasts from a healthy volunteer. (C) Hierarchical clustering and (D) principal component analysis of iPSCs and parental fibroblasts. (E) Heatmap of gene sets preferentially expressed in pluripotent stem cells. **(F)** Methylation analysis of *OCT3/4* and *NANOG* promotor regions. Color boxes indicate methylated while white boxes indicate demethylated allele. **(G)** Karyotype analysis of iPSCs. **(H)** Teratoma formation assay showing successful differentiation of iPSCs into three germ layers. **(I)** The results of the quantitative RT-PCR analyses of *OCT3/4*, *SOX2* and *L-MYC* expression in control and SMA-iPSCs relative to hESCs (KhES1). ‘‘Tg’’ indicates primers detecting the transgene only, whereas ‘‘total’’ indicates primers detecting both the endogenous gene and the transgene. Scale bars: 100 μm. The data are the means ±SEM of triplicate samples.

Figure S2: Characteristics of iPSC-derived MNs and AChR clusters, related to Figure 2

(A) Immunostaining of SMA-iPSC-derived MNs. MAP2 (red) and Tuj1 (green) stained on day 40. **(B)** Quantitative RT-PCR analysis of the *OCT3/4* expression in the PSCs and PSC-derived MNs. The data are presented as the mean ±SEM of triplicate samples. **(C)** Processed black and white images used for automatic area counting of neurons and AChR clustering. NF+SV2 indicates neuron-positive areas and αBTX indicates AChR-clustering positive areas. **(D)** Quantitative analysis of the size of each AChR cluster. (means ±SEM, n=3, Wilcoxon rank-sum test). **(E)** Quantitative analysis of the number of AChR clusters. (means ±SEM, n=3, Wilcoxon rank-sum test). **(F)** Quantitative RT-PCR analysis of the expression of *Myh8* and *Myh3* genes in differentiated C2C12 myotubes. The ratio of *Myh8* to *Myh3* is shown. The data are presented as the mean ±SEM of triplicate dishes. Neurobasal medium; monocultured C2C12 cells were with co-culturing medium. DMEM+FCS; monocultured C2C12 cells with their maintenance medium. **(G)** The representative immunostaining image of iPSC-derived MNs with TUNEL (green) and ChAT (red) and **(H)** their quantification. The data are presented as the mean ±SEM of triplicate dishes.

Figure S3: Effect of VPA and PMO treatment on AChR clustering, related to Figure 3 and Figure 4.

(A) Processed black and white images used for automatic area counting of neurons and AChR clustering with or without VPA treatment. **(B)** Summary of RNA-seq analysis. **(C)** Expression levels of splicing factors (*SF2/ASF, Htra2-β1, SRp20, hnRNPA1*) with VPA treatment. Fold change to the expression levels without VPA treatment are shown. **(D)** Processed black and white images used for automatic area counting of neurons and AChR clustering after PMO treatment.

Table S1: list of genes upregulated by VPA treatment (>2 fold to compared to untreated), related to Figure 3.

TRIM34+TRIM6+TRIM6-TRIM34	CALCB	ARNTL
TMC6+TMC8	FGFR4	PPIC
ALS2CL	NCEH1	GSN
ETHE1	TMEM176B	SEMA3C
BLVRB	MITF	WNT16
FLJ46906	PIK3R5	EPOR
PPFIBP2	PPP1R1B	MFAP2
PTPN3	CACNA1G	SMYD3
NRGN	RTBDN	MUM1L1
PAQR5	RIT2	TAC3
AMBN	AKR1C3	TMEM255A
DUX2+DUX4+DUX4L2+DUX4L3+DUX4L5+DUX4L6+DUX4L7	CXorf57	STEAP1
PAMR1	PENK	CRABP2
FSTL5	EGFL7	DENND2C
HOXD8	PLEKHB1	CAV2
DLEU1	GPR50	THEMIS2
CCDC152+SEPP1	NMNAT3	ALPL
CPT1A	ITM2A	TMCC3
MT2A	GLYATL1	GXYLT2
TMEM74	DPYD+DPYD-AS1	HLA-B
HLA-C	GABRE	ATP8B3
PRSS12	RAB7L1	LGALS1
CREG1	HLA-F+HLA-F-AS1	DNAJC12
SLC35F2	KCNJ4	IFI6
FXYD1	HEPH	IGSF1
DHRS2	ABCB1	CDK18
C4orf32	LRAT	TRAM1L1
NPAS1	VAV3	MIR1324
IL17RA	GPR158+GPR158-AS1	UTS2
PLCL1	SNORD114-8	GPC5
PLCG2	SERPINI1	PLEKHH2
DAPL1	CHRM4	IRF6
LPA	KCNIP2+LOC100289509	DPP10
NMRK1	CHRDL1	SULT1C4
TSPAN33	C9orf135	STAT4
USH2A	TESC	PAIP2B
IL16+STARD5	PRKCB	ARHGAP18
PPAP2C	KCTD19+PLEKHG4	C19orf77
TNNT1	TMEM176A	PRKCQ
ENPP5	GABRD	PPM1J
RASGRP2	CRYM	LPCAT2
SNORD61	HOXB7	PRKCG
HAPLN4	PDGFRA	C1orf115
FBLN2	ADRA1A	GRAMD1C
ARHGAP26	HPCA+TMEM54	TFEB
COL6A2	HERC5	ULBP1
DUSP23	KCNH3	ANO5
CHRNB4	OSTF1	TEX15
HLA-DPA1+HLA-DPB1	RAB11FIP1	GALNT10
SPAG6	MT1F	MIR5586
C3orf52	TPD52L1	RXRG
CYP2J2	SLC17A7	CD163L1
GALP	GMPR	C1QTNF9B+C1QTNF9B-AS1
STEAP1B	C4orf33	SLC2A4
SELL	CRLF1	TINCR
CRHR2	SNORA74A	LOC650368
CLDN6	TSPAN15	ALDH1A1
TMPRSS15	SCPEP1	SUSD3
IL13RA2	EDA	MIR3193
CD40	CLGN	C17orf96
RGS10	H2AFJ	DGAT2
MIR548AQ+MIR548AR	CD74	STAC2
C1QL1	MRAP2	SLC18A3
ITGA5	SYNPR	MIR7-3HG
GPX3	MIR4324	RNF128
NDST4	PLA2G4A	RLN2
PCBP3	ABCD2	FAM19A4
HLA-DMB	FTCD	RBM11
COL9A2	IFI30	C10orf128
TNNI3	A4GALT	LOC100506013
MICB	SLC9A9	RASD2
LOC401074	ARID5A	FAM101B
LOC401074	IMPA2	NRTN
MIR4735	MGARP	CHST9
SCGN	GPR37	ANKRD20A19P
ITIH5	SPINK2	

Table S2: list of genes downregulated by VPA treatment (>2 fold to compared to untreated), related to Figure 3.

EFCAB2	HYI+SZT2	MYD88
LINC00261	C4A+C4B+LOC100293534	LRFN4
TM4SF1	OBSL1	CLEC18A
BGLAP+PMF1+PMF1-BGLAP	GDPD2	SNORD4B
SNORD38B	ATP1A2	SNORD21
LPL	MIR93	MIR568
SNORD115-11+SNORD115-29+SNORD115-36+SNORD115-43	SERPINH1	ACSS1
LANCL2	SLC27A3	SNORD88A
AIFM2+H2AFY2	C4A+C4B+LOC100293534	SNORD12C
GLYCTK+MIR135A1	ACTG2	SOCS3
CLEC18C	MARVELD1	HIST1H2BC
MLC1	PDGFRB	MIR219-2+MIR2964A
SNORA51	RCBTB2	FADS2
MOV10	MIRLET7D	TAGLN
GTF2IRD2	MIR4508	MIR1305
MIR3175	MIR106B	CLEC18B
GTF2IRD2P1	SLC16A14	GTF2IRD2B

Table S3: primer sequences for PCR analysis, related to Figure 1-4.

primer name	Sequences (5' to 3')
NANOG	CCA AAG GCA AAC AAC CCA CTT
	GAC CGG GAC CTT GTC TTC CT
GAPDH	ACC ACA GTC CAT GCC ATC AC
	TCC ACC ACC CTG TTG CTG TA
total *OCT3/4*	CCC CAG GGC CCC ATT TTG GTA CC
	ACC TCA GTT TGA ATG CAT GGG AGA GC
transgene *OCT3/4*	CAT TCA AAC TGA GGT AAG GG
	TAG CGT AAA AGG AGC AAC ATA G
total *SOX2*	TTC ACA TGT CCC AGC ACT ACC AGA
	TCA CAT GTG TGA GAG GGG CAG TGT GC
transgene *SOX2*	TTC ACA TGT CCC AGC ACT ACC AGA
	TTT GTT TGA CAG GAG CGA CAA T
total *L-MYC*	GCG AAC CCA AGA CCC AGG CCT GCT CC
	CAG GGG GTC TGC TCG CAC CGT GAT G
transgene *L-MYC*	GGC TGA GAA GAG GAT GGC TAC
	TTT GTT TGA CAG GAG CGA CAA T
SMN-FL	CAAAAAGAAGGAAGGTGCTCACATT
	GTGTCATTTAGTGCTGCTCTATGC
SMN-⊿7	CTTGATGATGCTGATGCTTTGGGAAG
	CTATGCCAGCATTTCCATATAATAGCCAG
GAPDH	GTGGACCTGACCTGCCGTCT
	GGAGGAGTGGGTGTCGCTGT
Myh3	TGAGATTGCAGGATCTGGTGG
	CTCATGCTGGGCTTTCCTGA
Myh8	GCCGGGAGGTTCACACCAAA
	AAACCCAGAGAGGCAAGTGA

Supplementary experimental procedures

Study approval

Use of human ESCs was approved by the Ministry of Education, Culture, Sports, Science and Technology of Japan (MEXT). The study plan for recombinant DNA research has been approved by recombinant DNA experiments safety committee of Kyoto University. An experimental protocol was approved by the Animal Research Committee of Center for iPS cell research and application, Kyoto University.

Cell lines

The following fibroblasts were obtained from the NIGMS Human Genetic Cell Repository at the Coriell Institute for Medical Research: [GM00232, GM03813]. Donors of both fibroblasts were described as having SMA type I. The iPSCs generated from the GM00232 and GM03813 fibroblasts were renamed CiRA00070 and CiRA00069, respectively, according to the institutional regulations. A human embryonic stem cell line, KhES1, was kindly provided by Dr. Hirofumi Suemori (Institute for Frontier Medical Sciences, Kyoto University, Kyoto, Japan). The human iPS cell lines, 201B7 and 409B2, were kindly provided by Dr. Shinya Yamanaka (Center for iPS Cell Research and Application, Kyoto University, Kyoto, Japan). The murine myoblast cell line, C2C12, was kindly provided by Dr. Atsuko Sehara-Fujisawa (Institute for Frontier Medical Sciences, Kyoto University, Kyoto, Japan).

Establishment of iPSCs

Episomal vectors encoding reprogramming factors (*OCT3/4, SOX2, KLF4, L-MYC, LIN28* and p53 shRNA) were transduced into fibroblasts on day 0 as described previously (Okita et al., 2011). Plasmids were kindly provided by Dr. Keisuke Okita (CiRA, Kyoto University).The transfected cells were reseeded onto feeder layers on day 7, and maintained in embryonic stem cell medium (ReproCELL). Around day 30, the iPSC colonies were picked up as usual.

RNA isolation and quantitative PCR

Total RNA extraction from cells was performed using the RNeasy Mini kit (QIAGEN). One microgram of total RNA was used for reverse transcription with the PrimeScript RT Master Mix (TaKaRa). The real-time PCR was performed with SYBR Premix Ex TaqII (TaKaRa) in triplicate using the StepOnePlus system (Applied Biosystems). *GAPDH* was used as an endogenous control. The primer sets used for the quantitative PCR assay are described in **Table S3**.

Teratoma formation

The iPSCs recovered from one 6 cm dish were injected subcutaneously into NOG mice (Central Institute for Experimental Animals). Tumors were dissected eight weeks after injection and were fixed with PBS containing 4% paraformaldehyde. Paraffin-embedded tissues were sliced and stained with hematoxylin and eosin. Slides were examined using a BIOREVO BZ-9000 system (KEYENCE).

Genotyping (PCR-RFLP)

The methods used to detect homozygous *SMN1* exon 7 and 8 deletions were described previously (van der Steege et al., 1995). In brief, the restriction enzymes Dra I and Dde I cleave the PCR products originating only from *SMN2*, enabling *SMN1*-derived PCR products to be distinguished from those of *SMN2*. The completely

digested band corresponds to the deletion of exons 7 or 8 in *SMN1*. The results of the PCR electrophoresis were analyzed by a bioanalyzer (Agilent)(Burrell et al., 2011).

Protein isolation and Western blot analysis

Cells were isolated, suspended in M-PER (Thermo SCIENTIFIC), supplemented with 1% protease inhibitor cocktail (Sigma) and centrifuged at 20,000 g for 15 min at 4℃. A total of 3.125 μg of protein extracted from each sample was separated on 10% SDS-polyacrylamide gels, transferred to a nitrocellulose membrane, probed with a primary antibody against SMN (1:20,000; BD #610647), followed by horseradish-peroxidase-conjugated secondary antibody (1:10,000; Cell Signaling), and then visualized using ECL chemiluminescence reagents (Amersham). As a control, membranes were stripped and re-probed for β-actin (1:1,000; Cell Signaling #4970S). Quantitative densitometry analysis was performed using ImageQuant LAS 4000 software.

Immunocytochemistry and microscopy

Cells were fixed and permeabilized for 30 min at room temperature in 4% paraformaldehyde and 0.2% TritonX-100, and incubated with Block Ace (DS PHARMA BIOMEDICAL) to prevent any non-specific binding before overnight incubation with primary antibodies at 4℃. The following day, secondary antibody incubations were performed for 1 hour with the appropriate species-specific antiserum coupled to either FITC, Cy3 (Jackson ImmunoResearch, 1/100) or Alexa594 conjugated α-bungarotoxin (α-BTX) (Invitrogen #B13423, 1/500). After staining nuclei with DAPI (1/1,000), the cells were mounted utilizing Vectashield (Vector laboratories) and imaged using a LV1000 confocal microscope (Olympus). All antibodies were diluted in Block Ace. The following primary antibodies were used at the indicated concentrations: neurofilament 160 kDa (Millipore #MAB5254, 1/1000), Tuj1 (Covance #MMS435P, 1/2000), MAP2 (Sigma #M2320, 1/1000), HB9 (DSHB #81.5C10, 1/100), ChAT (Millipore #AB144P, 1/200), anti-synaptic vesicle protein 2 (DSHB #SV2, 1/20), NF-H (abcam #ab4680, 1/5000), mAb35 (DSHB #mAb35, 1:1,000), MHC (Millipore #A4.1025, 1:1,000) and TUNEL (Promega, DeadEnd Fluorometric TUNEL System).

Quantification of the results of the immunocytochemical analysis

Samples were imaged under identical gain and exposure settings. Only motor neurons containing a nucleus were counted in order to avoid double counting from adjoining sections. To calculate the average number of HB9-positive motor neurons, 12 visual fields in each preparation from each condition were counted using the BIOREVO BZ-9000 system (KEYENCE).

For the co-cultured samples, eight visual fields were evaluated and assessed in each preparation. Images were obtained with a 10×objective lens. For measurements, individual images of AChR clusters were imported into the ImageJ software program and split into red (endplate) and green (neurite and motor nerve terminal) channels. Each endplate and corresponding motor nerve terminal was automatically outlined and calculated using the IN Cell Analyzer 2000 software program (GE Healthcare Life Sciences), and areas greater than 427 pixels were kept as targets.

Microarray

Total RNA was extracted and purified using RNA mini kit (QIAGEN) according to manufacturer’s instruction. Gene expression analysis was performed using SurePrint G3 Human Gene Expression 8 × 60K kit (G4851A; Agilent technologies). Cy3-labelled cDNA was synthesized from 100 ng of RNA using Genomic DNA Enzymatic Labeling

Kit (Agilent Technologies). Labelled cDNA was fragmented with Gene Expression Hybridization Kit (Agilent Technologies), followed by hybridization at 65˚C for 17 hrs. Images were acquired by DNA Microarray Scanner (Agilent Technologies). The data were normalized by 75 percentile shift using the GeneSpring GX 12.6.1 software (Agilent Technologies). Principle component analysis (PCA) was performed by R ver 3.1.0. Two-way hierarchical clustering analysis was performed using Ward's method based on Euclidean distance. Heatmap was illustrated by GeneSpring GX 12.6.1 using average method based on Euclidean distance.

DNA methylation analysis

Genomic DNA was extracted and purified using PureLink Genomic DNA Mini Kit (Invitrogen) according to manufacturer's instruction. One μg of genomic DNA was subjected to bisulfite conversion of unmethylated cytosines of genomic DNA into uracils with EZ DNA Methylation Gold Kit (ZYMO Research). Promoter regions of *POU5F1* and *NANOG* were amplified by PCR from approximately 10 ng of bisulfite-treated DNA using Ex-taq (TaKaRa). PCR condition for *POU5F1* promoter was: 1 cycle of 98°C for 1 min, followed by 45 cycles of 98°C for 10 s, 60°C for 30 s, and 72°C for 1 min. That for *NANOG* promoter was: 1 cycle of 95°C for 3 min, followed by 40 cycles of 95°C for 30s, 58°C for 30 s, and 72°C for 1 min. Primers were: GTTTTTAGAGTAGTTGGGATTATAG and AACCCACCCTTATAAATTCTCAATTA for *NANOG*; ATTTGTTTTTTGGGTAGTTAAAGGT and CCAACTATCTTCATCTTAATAACATCC for *POU5F1*. Illumina libraries were generated by NEBNext Ultra DNA Library Prep Kit (New England BioLabs), and sequenced in 250 cycles Paired-end mode of MiSeq. All sequence reads were extracted in FASTQ format using MiSeq Reporter v2.3.32. Mapping to canonical sequence of human genome (hg19) was performed by bismark v.0.7.7 (Krueger and Andrews, 2011).

RNA-seq

After depletion of ribosomal RNA by RiboZero Gold (Epicentre), we prepared libraries using the Illumina TruSeq Stranded Total RNA Sample Prep kit. The libraries were sequenced in 100 cycle Single-Read mode of HiSeq2500. All sequence reads were extracted in FASTQ format using BCL2FASTQ Conversion Software 1.8.4 in the CASAVA 1.8.2 pipeline. The sequence reads were mapped to hg19 reference genes, downloaded on 10th December 2012, using Tophat v2.0.8b (Kim et al., 2013) and quantified by RPKMforGenes (Ramskold et al., 2009), downloaded on 19th, October 2012. Gene Ontology analysis was used by David v6.7 (Huang da et al., 2009).

GSE accession numbers

Microarray and RNA-seq deposited in GEO database can be accessed with the GEO accession number GSE65470 and GSE65508, respectively.

Motor neuron differentiation and co-culture with C2C12

The iPSCs were dissociated into single cells and quickly re-aggregated in DFK 5% medium (DMEM/F12 medium supplemented with KSR, NEAA, 2-mercaptoethanol, L-Glutamate, SB431542, dorsomorphin and Y27632) (9000 cells/150 μl/well) using 96-well low cell-adhesion plates (Lipidure-coat U96w from Nunc)(Eiraku et al., 2008; Morizane et al., 2011) . From day 8, the cell aggregates were treated with Sonic hedgehog (100 ng/ml) and retinoic acid (1 μM) for 1 week (Wada et al., 2009). On day 20, the cell aggregates were plated onto poly-l-lysin/laminin-coated culture dishes in neurobasal medium (Gibco) supplemented with the neurotrophic factors GDNF, BDNF and NT3 (10 ng/ml, R & D Systems). The medium was changed every 3-4 days thereafter.

For the co-culture with neuronal cells, the fusion of C2C12 myoblasts was induced by switching to the differentiation medium (DMEM supplemented with horse serum). On day 4, the MNs that had differentiated from the iPSCs (differentiation days 34-54) were harvested and plated on the induced myotubes, and the medium was changed to Neurobasal medium containing neurotrophic factors (BDNF, GDNF, NT3; 10 ng/ml each). Thereafter, the cultures were fed every two days by changing half of the medium.

VPA treatment

Co-cultured samples were treated with or without 1 mM VPA by changing half of the medium every two days. After six days of drug treatment, the area of NF and αBTX immunostaining was detected by immunocytochemistry and was analyzed by the IN Cell Analyzer 2000 software program.

PMO treatment

Designed PMOs SMN2E7D(-10-29) for suppressing splice silencing motifs in intron 7 of *SMN2* (Mitrpant et al., 2013) and its negative control were purchased from GENE TOOLS. SMN- or Ctrl-PMO (10 µM in medium) were introduced with the Endo-Porter (GENE TOOLS) on day 1 of co-culturing, and the cells were subsequently cultured for three days.

Statistics. Statistic functions in Microsoft Excel 2013 was used for statistical analyses. Results are expressed as mean ± SEM. Statistical significance was determined using Student's t test and Wilcoxon rank sum test. $P<0.05$ was considered significant. 'n' represents the number of independent experiments.

Supplemental References

Burrell, A., Foy, C., and Burns, M. (2011). Applicability of three alternative instruments for food authenticity analysis: GMO identification. Biotechnology research international *2011*, 838232.

Eiraku, M., Watanabe, K., Matsuo-Takasaki, M., Kawada, M., Yonemura, S., Matsumura, M., Wataya, T., Nishiyama, A., Muguruma, K., and Sasai, Y. (2008). Self-organized formation of polarized cortical tissues from ESCs and its active manipulation by extrinsic signals. Cell stem cell *3*, 519-532.

Huang da, W., Sherman, B.T., and Lempicki, R.A. (2009). Systematic and integrative analysis of large gene lists using DAVID bioinformatics resources. Nat Protoc *4*, 44-57.

Kim, D., Pertea, G., Trapnell, C., Pimentel, H., Kelley, R., and Salzberg, S.L. (2013). TopHat2: accurate alignment of transcriptomes in the presence of insertions, deletions and gene fusions. Genome Biol *14*, R36.

Krueger, F., and Andrews, S.R. (2011). Bismark: a flexible aligner and methylation caller for Bisulfite-Seq applications. Bioinformatics *27*, 1571-1572.

Mitrpant, C., Porensky, P., Zhou, H., Price, L., Muntoni, F., Fletcher, S., Wilton, S.D., and Burghes, A.H. (2013). Improved antisense oligonucleotide design to suppress aberrant SMN2 gene transcript processing: towards a treatment for spinal muscular atrophy. PLoS One *8*, e62114.

Morizane, A., Doi, D., Kikuchi, T., Nishimura, K., and Takahashi, J. (2011). Small-molecule inhibitors of bone morphogenic protein and activin/nodal signals promote highly efficient neural induction from human pluripotent stem cells. J Neurosci Res *89*, 117-126.

Okita, K., Matsumura, Y., Sato, Y., Okada, A., Morizane, A., Okamoto, S., Hong, H., Nakagawa, M., Tanabe, K., Tezuka, K.*, et al.* (2011). A more efficient method to generate integration-free human iPS cells. Nat Methods *8*, 409-412.

Ramskold, D., Wang, E.T., Burge, C.B., and Sandberg, R. (2009). An abundance of ubiquitously expressed genes revealed by tissue transcriptome sequence data. PLoS Comput Biol *5*, e1000598.

van der Steege, G., Grootscholten, P.M., van der Vlies, P., Draaijers, T.G., Osinga, J., Cobben, J.M., Scheffer, H., and Buys, C.H. (1995). PCR-based DNA test to confirm clinical diagnosis of autosomal recessive spinal muscular atrophy. Lancet *345*, 985-986.

Wada, T., Honda, M., Minami, I., Tooi, N., Amagai, Y., Nakatsuji, N., and Aiba, K. (2009). Highly efficient differentiation and enrichment of spinal motor neurons derived from human and monkey embryonic stem cells. PloS one *4*, e6722.
